# Expression of feeding-related neuromodulatory signalling molecules in the mouse central olfactory system

**DOI:** 10.1038/s41598-020-57605-7

**Published:** 2020-01-21

**Authors:** Yasuko Nogi, Md Monjurul Ahasan, Yoshihiro Murata, Mutsuo Taniguchi, Md Fazley Rabbi Sha, Chiori Ijichi, Masahiro Yamaguchi

**Affiliations:** 1 Institute of Food Sciences and Technologies, Ajinomoto Co., Inc., Kanagawa, Japan; 2 Department of Physiology, Kochi Medical School, Kochi University, Kochi, Japan

**Keywords:** Feeding behaviour, Molecular neuroscience, Neural circuits, Olfactory cortex

## Abstract

Various neural systems cooperate in feeding behaviour, and olfaction plays crucial roles in detecting and evaluating food objects. While odour-mediated feeding behaviour is highly adaptive and influenced by metabolic state, hedonic cues and learning processes, the underlying mechanism is not well understood. Feeding behaviour is regulated by orexigenic and anorexigenic neuromodulatory molecules. However, knowledge of their roles especially in higher olfactory areas is limited. Given the potentiation of feeding behaviour in hunger state, we systemically examined the expression of feeding-related neuromodulatory molecules in food-restricted mice through quantitative PCR, in the olfactory bulb (OB), olfactory tubercle (OT), and remaining olfactory cortical area (OC). The OT was further divided into attraction-related anteromedial, aversion-related lateral and remaining central regions. Examination of 23 molecules including neuropeptides, opioids, cannabinoids, and their receptors as well as signalling molecules showed that they had different expression patterns, with many showing elevated expression in the OT, especially in the anteromedial and central OT. Further, in mice trained with odour-food association, the expression was significantly altered and the increase or decrease of a given molecule varied among areas. These results suggest that different olfactory areas are regulated separately by feeding-related molecules, which contributes to the adaptive regulation of feeding behaviour.

## Introduction

To support growth and maintenance of homeostasis, animals obtain energy and nutrition through feeding. Feeding behaviour is therefore strongly regulated by metabolic status. In addition, feeding behaviour is regulated by the motivation to obtain pleasure from palatable food. These homeostatic and hedonic regulation strategies are regarded as key components of the mechanism driving feeding behaviour^1–4^.

In line with those strategies, a variety of functional systems in the brain and body work together to enable feeding; in particular, sensory systems in the brain are highly involved. Animals search for food via odour cues, and judge whether to accept or reject objects by integrating odour, taste and oral somatosensory cues^5^. In accordance with its pivotal role in feeding, olfaction is known to be influenced by metabolic state. Odour detection ability is enhanced during fasting, and conversely diminished with satiety in both rodents and humans^6,7^. In addition, olfaction strongly drives motivated behaviours, and feeding is a typical odour-mediated motivated and hedonic behaviour^8,9^. Further, odour-mediated behaviours are shaped by experience. Animals continually encounter novel food objects in the environment, and it has been argued that the odour-based edibility of food objects is mostly learned through experience, while a large proportion of taste-based edibility discriminations are innate^10^. Thus, the olfactory system has highly adaptive properties and acts as an interface linking external information about food objects with internal information about metabolic state, hedonic value and experience, to achieve proper feeding behaviour.

Feeding-related neuromodulators are a group of molecules that regulate neuronal activity, reflecting metabolic state and hedonic value, and also contribute to learning and memory^11^. Numerous feeding-related neuromodulatory signalling molecules are expressed in the olfactory system. These molecules are categorized as orexigenic or anorexigenic mainly based on their activity in the hypothalamus^4,12^, and a variety of orexigenic and anorexigenic neuropeptides, endogenous opioids, cannabinoids and their receptors are expressed in the olfactory system^13^. It is important to understand how these molecules are expressed and function in the olfactory system, and some studies have addressed their roles in odour information processing and odour-mediated feeding behaviour^13,14^. However, most analyses have been conducted in the olfactory epithelium and olfactory bulb (OB), which are the initial two structures for odour information processing. Because odour information is further transferred to cortical centres, it is also important to understand how feeding-related neuromodulatory molecules are expressed in higher olfactory areas, and how these areas contribute to the regulation of odour-mediated feeding behaviours.

The olfactory cortex can be divided into several structures including the anterior olfactory nucleus, olfactory tubercle (OT), piriform cortex, cortical amygdala and entorhinal cortex^15^. Among the various parts of the olfactory cortex, the OT is a unique area, in that it contains medium spiny neurons as its principal neurons and receives massive dopaminergic inputs from the midbrain^16–18^. The OT constitutes the ventral striatum with nucleus accumbens, and is considered a key structure mediating odour-induced motivated behaviours^19–21^. We previously showed that the OT has domain structures representing various types of odour-induced motivated behaviours. Mice that associate a neutral odour with a food reward become attracted to the learned odour, and presentation of that odour activates the anteromedial domain of the OT. In contrast, mice that associate the same odour with a foot shock punishment show aversive behaviour to the learned odour, and presentation of the odour activates the lateral domain of the OT^22^. Considering the differential roles of the anteromedial and lateral OT, these two domains might be expected to show differential expression of feeding-related neuromodulatory molecules, and may be differentially regulated by such molecules.

Based on this background, we examined the expression of feeding-related molecules in various olfactory regions of the mouse brain, including the OB, the two domains of the OT and other olfactory cortical areas, and further examined whether expression changes adaptively when mice are trained with odour-food association. Given the effectiveness of odour-food association under hunger state^8^, we focused our analyses on food-restricted mice and examined adaptive properties of feeding-related molecule expression with or without odour-food association training. We selected 23 feeding-related neuromodulatory signalling molecules for examination by quantitative PCR, and found that many of them showed differential expression among different olfactory areas and altered expression after odour-food association training.

## Results

### Dissection of olfactory areas of food-restricted mice with or without odour-food reward association training

**Figure 1:**
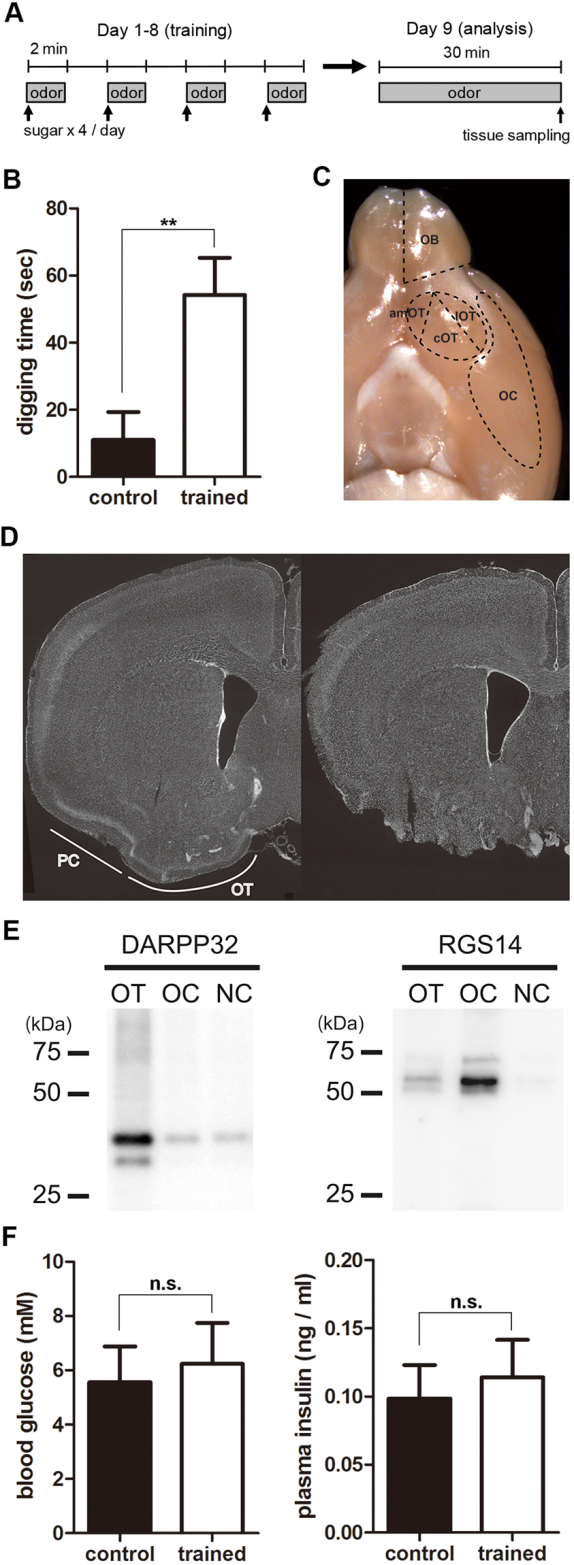
Odour-food reward association training and dissection of olfactory areas. (**A**) Protocol for odour-food association training. For trained mice, food was restricted and odour (eugenol) was presented with sugar four times per day from day 1 to day 8. On day 9, the mice were presented with the odour but no sugar for 30 min, and their digging behaviour over the odour stimulus was evaluated. Control mice were subjected to the same food restriction, odour presentation without sugar from day 1 to day 8, and behavioural analysis on day 9. (**B**) Digging behaviour of control and trained mice. On day 9, the digging behaviour of mice over the odour stimulus during the 30-min test period was evaluated for control and trained mice. Data show the average ± SEM. n = 5 for each treatment; **p < 0.01 (t-test). (**C**) Dissection of olfactory areas from the mouse brain. The ventral view of the mouse brain is shown. The olfactory bulb (OB), three areas of the olfactory tubercle (amOT, cOT, lOT), and olfactory cortical area (OC) were dissected as indicated. (**D**) Histological examination of the OT and OC dissection. Coronal sections of intact brain (left) and dissected brain (right) were stained with DAPI. The OT and piriform cortex (PC) were appropriately dissected. (**E**) Molecular expression of dissected olfactory areas. Protein extracts from the OT, OC, and neocortical area (NC) were electrophoresed and examined for DARPP32 (left) and RGS14 (right) expression using immunoblots. Images of blots have been cropped and full images are included in the Supplementary Information. (**F**) Blood glucose and plasma insulin concentrations in control and trained mice. After behavioural tests on day 9, the blood was collected and the blood glucose (left) and plasma insulin (right) were measured. Data show the average ± SEM. n = 6 for control and 5 for trained groups. n.s., not significant (t-test).

To examine potential changes in feeding-related neuromodulatory molecule expression with odour-food association, mice were divided into a group trained to associate a particular odour with a food reward and a control group. C57BL/6 male mice at 8 weeks of age (n = 9 mice) were food restricted to 80–90% of their original body weight, and were presented with an odour molecule, eugenol, combined with a sugar reward four times per day (Fig. 1A). Control mice (n = 9) were similarly food-restricted and presented with eugenol alone. This procedure was conducted for 8 successive days. On day 8, all the trained mice located the eugenol-scented sugar under the bedding very rapidly. Quantification of the behaviour from randomly sampled mice (n = 5 mice) showed their short latency for odour source detection (latency on the first trial, 5.8 ± 1.6 s; latency on the fourth trial, 3.0 ± 0.7 s; average ± SEM). On the other hand, no control mice dug into the bedding over the eugenol during the 2-min observation period.

On day 9, mice were presented with eugenol alone without a sugar reward under the bedding. All the trained mice showed extensive food searching behaviour toward the odour source. Quantification of the digging behaviour over the odour source during the 30-min test period for the sampled mice (n = 5 for each group) showed that trained mice extensively dug the bedding over the odour stimulus to search for the sugar reward, while control mice showed only a short period of digging behaviour over the odour stimulus (Fig. 1B). Digging behaviour of trained mice was observed within 10 seconds after their introduction into the test cage, while that in control mice was delayed by least several minutes (data not shown). These behaviours clearly showed that trained mice associated the odour cue with a food reward, while control mice did not.

After the 30-min test period, mice were deeply anesthetized and sacrificed, and the brain tissues were recovered. The OB, OT, and olfactory cortical area except the OT (referred to as OC; major structure was likely the piriform cortex) were dissected (Fig. 1C). Figure 1D shows the remaining brain structure after dissection of the olfactory areas. The OT and piriform cortex were removed, while deeper brain structures remained intact. Western blot analysis showed that DARPP-32, a signalling molecule downstream of dopamine receptors^23^, was enriched in the OT sample (Fig. 1E, left). RGS14, a regulator of G-protein signalling that is highly expressed in the piriform cortex and hippocampus^24^, was enriched in the OC sample but not in the OT or neocortex (NC) sample (Fig. 1E, right). These results confirmed the proper dissection of olfactory areas.

The OT contains functional domains, with the anteromedial domain being responsible for attractive behaviour and the lateral domain for aversive behaviour^22^. Thus, the OT was further divided into the anteromedial region (amOT), lateral region (lOT) and remaining central region (cOT) (Fig. 1C; see Methods). Accordingly, the five olfactory areas of OB, OC, amOT, cOT, and lOT were each collected from control and trained mice, and then processed for mRNA expression analysis.

Trained mice were supplied with small amount of sugar reward during 8 training days (~200 mg per day). To examine whether the sugar supply altered the metabolic state of mice, glucose and insulin contents in the peripheral blood were examined in different sets of mice that received same food restriction and training processes as described above. On day 8, all the trained mice (n = 5) located the eugenol-scented sugar under the bedding very rapidly (latency on the first trial, 4.8 ± 1.5 s; latency on the fourth trial, 3.0 ± 1.2 s; average ± SEM), while control mice (n = 6) did not during the 2-min observation period. On day 9, trained mice but not control mice exhibited extensive digging behaviour over the odour source during the 30-min test period (trained mice, 44.4 ± 7.8 s, n = 5; control mice, 6.2 ± 2.9 s, n = 6; average ± SEM). After the behaviour test they were deeply anesthetized and sacrificed, and their blood was collected. The blood glucose and plasma insulin concentrations did not differ between the two groups (Fig. 1F), suggesting that the metabolic state of trained mice was not significantly altered by the sugar supply.

### Expression of feeding-related neuromodulatory molecules in different olfactory areas of control mice

mRNA collected from the olfactory areas was subjected to quantitative real-time PCR to determine the expression levels of feeding-related neuromodulatory molecules. We selected 23 molecules related to feeding, including neuropeptides, growth factors, endogenous opioids, cannabinoids, and their receptors, as well as related signalling molecules (Table 1). The expression levels were normalized to that of the housekeeping gene glyceraldehyde-3-phosphate dehydrogenase (Gapdh), which was used as the internal control because its expression level did not differ significantly among the five olfactory areas based on quantitative real-time PCR (data not shown).

**Table 1:** Neuromodulatory signalling molecules examined and PCR probes used in this study.

Gene Symbol	Gene Name	TaqMan® Gene Expression Assays
Avp	arginine vasopressin	Mm00437761_g1
Avpr1a	arginine vasopressin receptor 1A	Mm00444092_m1
Bdnf	brain derived neurotrophic factor	Mm04230607_s1
Cartpt	CART prepropeptide	Mm04210469_m1
Cnr1	cannabinoid receptor 1 (brain)	Mm01212171_s1
Cnr2	cannabinoid receptor 2 (macrophage)	Mm02620087_s1
Gapdh	glyceraldehyde-3-phosphate dehydrogenase	Mm99999915_g1
Ghrl	ghrelin	Mm00612524_m1
Ghsr	growth hormone secretagogue receptor	Mm00616415_m1
Hcrt	hypocretin	Mm01964030_s1
Hcrtr1	hypocretin (orexin) receptor 1	Mm01185776_m1
Hcrtr2	hypocretin (orexin) receptor 2	Mm01179312_m1
Lepr	leptin receptor	Mm00440181_m1
Mc4r	melanocortin 4 receptor	Mm00457483_s1
Mme	membrane metallo endopeptidase	Mm01285052_m1
Npy2r	neuropeptide Y receptor Y2	Mm01956783_s1
Oprd1	opioid receptor, delta 1	Mm01180757_m1
Oprk1	opioid receptor, kappa 1	Mm01230885_m1
Oxt	oxytocin	Mm01329577_g1
Oxtr	oxytocin receptor	Mm01182684_m1
Pdyn	prodynorphin	Mm00457573_m1
Penk	preproenkephalin	Mm01212875_m1
Pomc	pro-opiomelanocortin-alpha	Mm00435874_m1
Tac1	tachykinin 1	Mm01166996_m1

**Figure 2:**
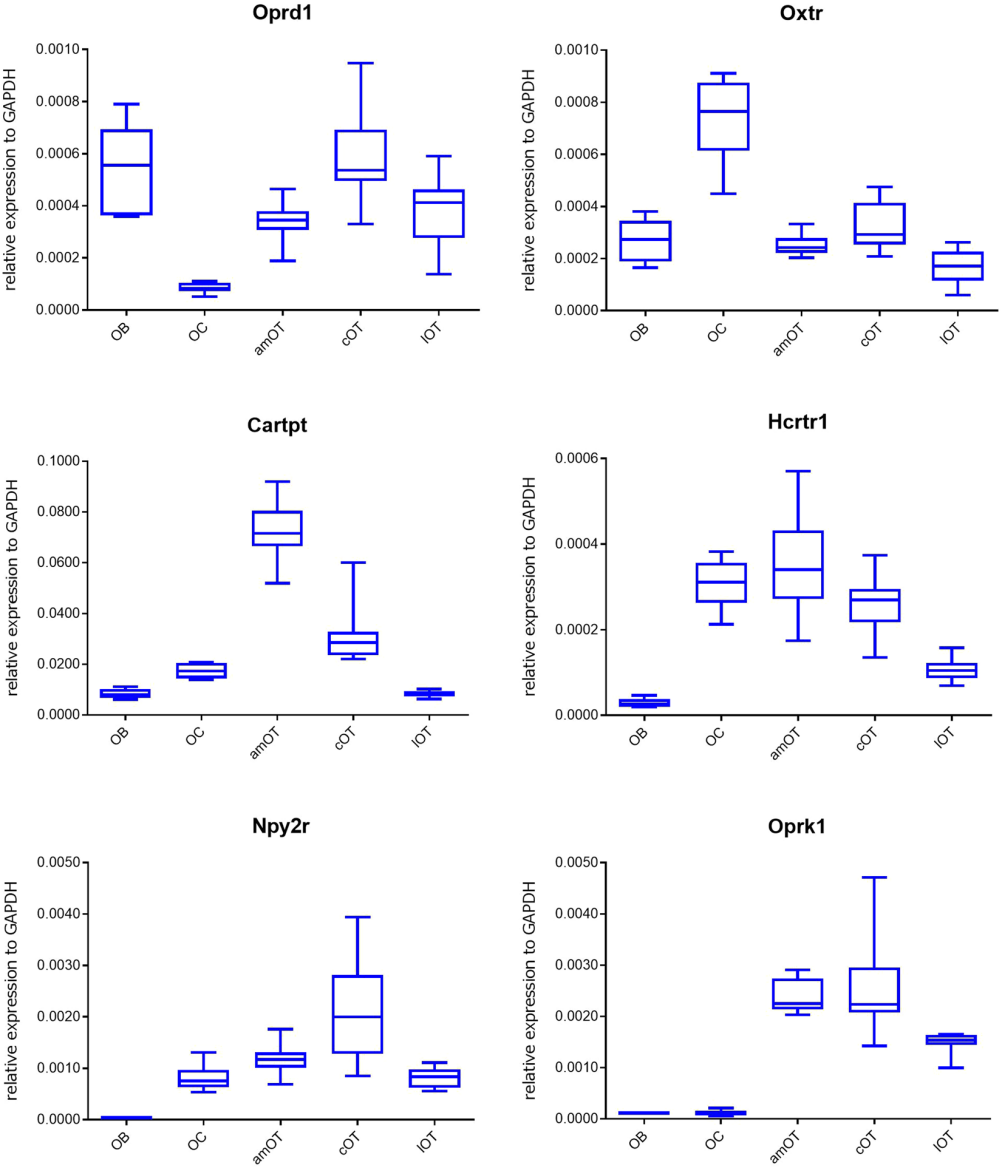
Boxplot analysis of representative neuromodulatory molecule expression in various olfactory areas of control mice. Data for opioid receptor delta 1 (Oprd1), oxytocin receptor (Oxtr), CART peptide (Cartpt), orexin receptor 1 (Hcrtr1), neuropeptide Y receptor type 2 (Npy2r) and opioid receptor kappa 1 (Oprk1) are shown. Boxes indicate the 25^th^ and 75^th^ percentiles, whiskers show the minimum and maximum values, and lines inside the boxes indicate the median. n = 9 in each group.

Firstly, expression levels in different olfactory areas were compared in control mice, which were subjected to food restriction and odour (eugenol) presentation that was not associated with a food reward. The data are shown in Table 2 as mean ± SD, in Supplementary Table S1 as F and p values, in Table 3 as post-hoc Tukey-Kramer comparison tests, and as representative figures in Fig. 2, and figures for all 23 molecules in Supplementary Fig. S1. In each group of data, 7–9 samples from different mice were examined.

**Table 2:** Expression of neuromodulatory molecules in individual olfactory areas of control mice. Data show the amount of each molecule relative to Gapdh (×10^−3^; mean ± standard deviation (SD)).

	OB	OC	amOT	cOT	lOT
Avp	0.02 ± 0.01	0.08 ± 0.04	0.25 ± 0.19	0.42 ± 0.27	0.16 ± 0.10
Avpr1a	0.15 ± 0.04	0.06 ± 0.02	1.10 ± 0.46	0.72 ± 0.19	0.26 ± 0.08
Bdnf	1.51 ± 0.22	4.91 ± 0.81	0.24 ± 0.18	0.14 ± 0.10	0.16 ± 0.17
Cartpt	8.50 ± 1.85	17.56 ± 2.95	72.73 ± 11.50	31.15 ± 11.53	8.55 ± 1.27
Cnr1	2.75 ± 0.42	14.42 ± 2.38	13.31 ± 2.41	17.23 ± 3.89	15.28 ± 1.99
Cnr2	0.16 ± 0.03	0.13 ± 0.02	0.14 ± 0.04	0.31 ± 0.07	0.16 ± 0.02
Ghrl	0.17 ± 0.05	0.08 ± 0.02	0.08 ± 0.04	0.24 ± 0.08	0.07 ± 0.04
Ghsr	0.16 ± 0.05	0.18 ± 0.12	0.27 ± 0.06	0.24 ± 0.05	0.06 ± 0.02
Hcrt	0.03 ± 0.01	0.05 ± 0.01	0.05 ± 0.01	0.06 ± 0.02	0.03 ± 0.01
Hcrtr1	0.03 ± 0.01	0.31 ± 0.06	0.35 ± 0.12	0.26 ± 0.07	0.11 ± 0.03
Hcrtr2	0.02 ± 0.01	0.16 ± 0.03	0.37 ± 0.06	0.38 ± 0.09	0.27 ± 0.04
Lepr	0.51 ± 0.10	0.60 ± 0.08	0.92 ± 0.37	1.03 ± 0.42	0.72 ± 0.17
Mc4r	0.02 ± 0.00	0.21 ± 0.07	2.57 ± 0.67	3.78 ± 1.02	1.46 ± 0.26
Mme	0.42 ± 0.12	0.21 ± 0.05	3.37 ± 0.95	7.55 ± 2.64	7.97 ± 1.21
Npy2r	0.02 ± 0.01	0.80 ± 0.25	1.17 ± 0.29	2.11 ± 1.02	0.82 ± 0.20
Oprd1	0.53 ± 0.17	0.09 ± 0.02	0.34 ± 0.07	0.59 ± 0.17	0.37 ± 0.14
Oprk1	0.12 ± 0.02	0.12 ± 0.05	2.39 ± 0.32	2.56 ± 0.96	1.49 ± 0.21
Oxt	0.01 ± 0.00	0.01 ± 0.01	0.07 ± 0.05	0.13 ± 0.14	0.03 ± 0.05
Oxtr	0.27 ± 0.08	0.74 ± 0.16	0.25 ± 0.04	0.33 ± 0.09	0.17 ± 0.07
Pdyn	0.55 ± 0.09	1.59 ± 0.35	41.92 ± 9.23	46.67 ± 15.63	27.85 ± 6.05
Penk	67.54 ± 4.37	31.60 ± 3.96	349.16 ± 42.85	449.16 ± 149.10	302.22 ± 35.55
Pomc	0.46 ± 0.62	1.31 ± 2.05	2.16 ± 3.69	1.31 ± 1.02	2.24 ± 2.81
Tac1	0.67 ± 0.12	1.24 ± 0.37	71.76 ± 12.70	96.19 ± 29.35	68.58 ± 7.17

**Table 3:** Post-hoc Tukey-Kramer comparison of molecular expression among five olfactory areas of control mice. Bold letters indicate significant p values (p < 0.05).

Compared pair	Avp	Avpr1a	Bdnf	Cartpt
OB-OC	*p* = 0.9180	*p* = 0.9072	*p* < **0.0001**	*p* = 0.0951
OB-amOT	*p* = **0.0291**	*p* < **0.0001**	*p* < **0.0001**	*p* < **0.0001**
OB-cOT	*p* < **0.0001**	*p* < **0.0001**	*p* < **0.0001**	*p* < **0.0001**
OB-lOT	*p* = 0.3290	*p* = 0.8462	*p* < **0.0001**	*p* = 1.0000
OC-amOT	*p* = 0.1852	*p* < **0.0001**	*p* < **0.0001**	*p* < **0.0001**
OC-cOT	*p* = **0.0004**	*p* < **0.0001**	*p* < **0.0001**	*p* = **0.0035**
OC-lOT	*p* = 0.8184	*p* = 0.3425	*p* < **0.0001**	*p* = 0.0982
amOT-cOT	*p* = 0.1549	*p* = **0.0102**	*p* = 0.9860	*p* < **0.0001**
amOT-lOT	*p* = 0.7689	*p* < **0.0001**	*p* = 0.9938	*p* < **0.0001**
cOT-lOT	*p* = **0.0095**	*p* = **0.0009**	*p* = 1.0000	*p* < **0.0001**
**Compared pair**	**Cnr1**	**Cnr2**	**Ghrl**	**Ghsr**
OB-OC	*p* < **0.0001**	*p* = 0.4798	*p* = **0.0097**	*p* = 0.9534
OB-amOT	*p* < **0.0001**	*p* = 0.6372	*p* = **0.0099**	*p* = **0.0158**
OB-cOT	*p* < **0.0001**	*p* < **0.0001**	*p* = 0.0900	*p* = 0.1206
OB-lOT	*p* < **0.0001**	*p* = 1.0000	*p* = **0.0020**	*p* = **0.0474**
OC-amOT	*p* = 0.8740	*p* = 0.9991	*p* = 1.0000	*p* = 0.0866
OC-cOT	*p* = 0.1342	*p* < **0.0001**	*p* < **0.0001**	*p* = 0.3875
OC-lOT	*p* = 0.9475	*p* = 0.5186	*p* = 0.9755	*p* = **0.0078**
amOT-cOT	*p* = **0.0140**	*p* < **0.0001**	*p* < **0.0001**	*p* = 0.9656
amOT-lOT	*p* = 0.4537	*p* = 0.6761	*p* = 0.9744	*p* < **0.0001**
cOT-lOT	*p* = 0.4602	*p* < **0.0001**	*p* < **0.0001**	*p* < **0.0001**
**Compared pair**	**Hcrt**	**Hcrtr1**	**Hcrtr2**	**Lepr**
OB-OC	*p* = **0.0249**	*p* < **0.0001**	*p* < **0.0001**	*p* = 0.9596
OB-amOT	*p* = **0.0029**	*p* < **0.0001**	*p* < **0.0001**	*p* = **0.0184**
OB-cOT	*p* < **0.0001**	*p* < **0.0001**	*p* < **0.0001**	*p* = **0.0018**
OB-lOT	*p* = 0.9959	*p* = 0.1287	*p* < **0.0001**	*p* = 0.4625
OC-amOT	*p* = 0.9339	*p* = 0.6499	*p* < **0.0001**	*p* = 0.0939
OC-cOT	*p* = 0.2129	*p* = 0.5529	*p* < **0.0001**	*p* = **0.0120**
OC-lOT	*p* = **0.0094**	*p* < **0.0001**	*p* = **0.0008**	*p* = 0.8576
amOT-cOT	*p* = 0.6412	*p* = **0.0456**	*p* = 0.9809	*p* = 0.9158
amOT-lOT	*p* = **0.0010**	*p* < **0.0001**	*p* = **0.0009**	*p* = 0.5142
cOT-lOT	*p* < **0.0001**	*p* = **0.0003**	*p* = **0.0002**	*p* = 0.1285
**Compared pair**	**Mc4r**	**Mme**	**Npy2r**	**Oprd1**
OB-OC	*p* = 0.9494	*p* = 0.9976	*p* = **0.0148**	*p* < **0.0001**
OB-amOT	*p* < **0.0001**	*p* = **0.0004**	*p* = **0.0001**	*p* = **0.0211**
OB-cOT	*p* < **0.0001**	*p* < **0.0001**	*p* < **0.0001**	*p* = 0.8884
OB-lOT	*p* < **0.0001**	*p* < **0.0001**	*p* = **0.0124**	*p* = 0.0729
OC-amOT	*p* < **0.0001**	*p* = **0.0002**	*p* = 0.5079	*p* = **0.0017**
OC-cOT	*p* < **0.0001**	*p* < **0.0001**	*p* < **0.0001**	*p* < **0.0001**
OC-lOT	*p* = **0.0003**	*p* < **0.0001**	*p* = 1.0000	*p* = **0.0004**
amOT-cOT	*p* = **0.0004**	*p* < **0.0001**	*p* = **0.0022**	*p* = **0.0016**
amOT-lOT	*p* = **0.0013**	*p* < **0.0001**	*p* = 0.5512	*p* = 0.9859
cOT-lOT	*p* < **0.0001**	*p* = 0.9647	*p* < **0.0001**	*p* = **0.0071**
**Compared pair**	**Oprk1**	**Oxt**	**Oxtr**	**Pdyn**
OB-OC	*p* = 1.0000	*p* = 1.0000	*p* < **0.0001**	*p* = 0.9990
OB-amOT	*p* < **0.0001**	*p* = 0.4645	*p* = 0.9937	*p* < **0.0001**
OB-cOT	*p* < **0.0001**	*p* = **0.0196**	*p* = 0.6918	*p* < **0.0001**
OB-lOT	*p* < **0.0001**	*p* = 0.9528	*p* = 0.1859	*p* < **0.0001**
OC-amOT	*p* < **0.0001**	*p* = 0.4249	*p* < **0.0001**	*p* < **0.0001**
OC-cOT	*p* < **0.0001**	*p* = **0.0124**	*p* < **0.0001**	*p* < **0.0001**
OC-lOT	*p* < **0.0001**	*p* = 0.9526	*p* < **0.0001**	*p* < **0.0001**
amOT-cOT	*p* = 0.9267	*p* = 0.4562	*p* = 0.4369	*p* = 0.7641
amOT-lOT	*p* = **0.0017**	*p* = 0.8423	*p* = 0.3724	*p* = **0.0100**
cOT-lOT	*p* = **0.0002**	*p* = 0.0713	*p* = **0.0087**	*p* = **0.0003**
**Compared pair**	**Penk**	**Pomc**	**Tac1**	
OB-OC	*p* = 0.8205	*p* = 0.9494	*p* = 1.0000	
OB-amOT	*p* < **0.0001**	*p* = 0.5981	*p* < **0.0001**	
OB-cOT	*p* < **0.0001**	*p* = 0.9487	*p* < **0.0001**	
OB-lOT	*p* < **0.0001**	*p* = 0.5535	*p* < **0.0001**	
OC-amOT	*p* < **0.0001**	*p* = 0.9481	*p* < **0.0001**	
OC-cOT	*p* < **0.0001**	*p* = 1.0000	*p* < **0.0001**	
OC-lOT	*p* < **0.0001**	*p* = 0.9285	*p* < **0.0001**	
amOT-cOT	*p* = **0.0372**	*p* = 0.9488	*p* = **0.0087**	
amOT-lOT	*p* = 0.6324	*p* = 1.0000	*p* = 0.9905	
cOT-lOT	*p* = **0.0008**	*p* = 0.9293	*p* = **0.0024**

Most molecules showed differing expression levels among olfactory areas. Generally, expression was relatively high in the OC and OT compared to the OB. For most of the 23 molecules examined, expression in the OC and/or OT was higher than or equivalent to that in the OB (Tables 2 and 3, Supplementary Fig. S1). Only ghrelin (Ghrl) and opioid receptor delta 1 (Oprd1) showed higher expression in the OB compared to the OC (see Fig. 2 for Oprd1 expression).

Further, many molecules showed higher expression in the OT than in the OB and OC. Orexin receptor 2 (Hcrt2), melanocortin 4 receptor (Mc4r), neprilysin (Mme), opioid receptor kappa 1 (Oprk1), prodynorphin (Pdyn), preproenkephalin (Penk) and tachykinin 1 (Tac1) showed higher expression in all three OT areas than in the OB or OC. A few molecules, such as brain-derived neurotrophic factor (Bdnf) and oxytocin receptor (Oxtr), had their highest expression levels in the OC (see Fig. 2 for Oxtr expression). Among the three OT areas, many molecules showed higher expression in the anteromedial and central OT. Expression levels of arginine-vasopressin receptor 1a (AVP1a), CART peptide (Cartpt) and orexin receptor 1 (Hcrtr1) were highest in the anteromedial OT among the three OT areas (see Fig. 2 for Cartpt and Hcrtr1 expression). Expression levels of cannabinoid receptor 2 (Cnr2), ghrelin (Ghrl), melanocortin 4 receptor (Mc4r), neuropeptide Y receptor type 2 (Npy2r), opioid receptor delta 1 (Oprd1), preproenkephalin (Penk) and tachykinin 1 (Tac1) were highest in the central OT (see Fig. 2 for Npy2r expression). Expression levels of ghrelin receptor (Ghsr), orexin (Hcrt), orexin receptor 2 (Hcrtr2), opioid receptor kappa 1 (Oprk1) and prodynorphin (Pdyn) were higher in the anteromedial and central OT than in the lateral OT (see Fig. 2 for Oprk1 expression). Conversely, neprilysin (Mme) showed higher expression in the lateral OT than in the anteromedial OT.

Taken together, while each neuromodulatory molecule examined in this study had differing expression profiles among olfactory areas, the overall tendency was that many molecules showed higher expression in the OT than in the OB and OC, especially in the anteromedial and central OT.

### Differential expression of neuromodulatory molecules between odour-food reward association-trained mice and control mice

**Figure 3:**
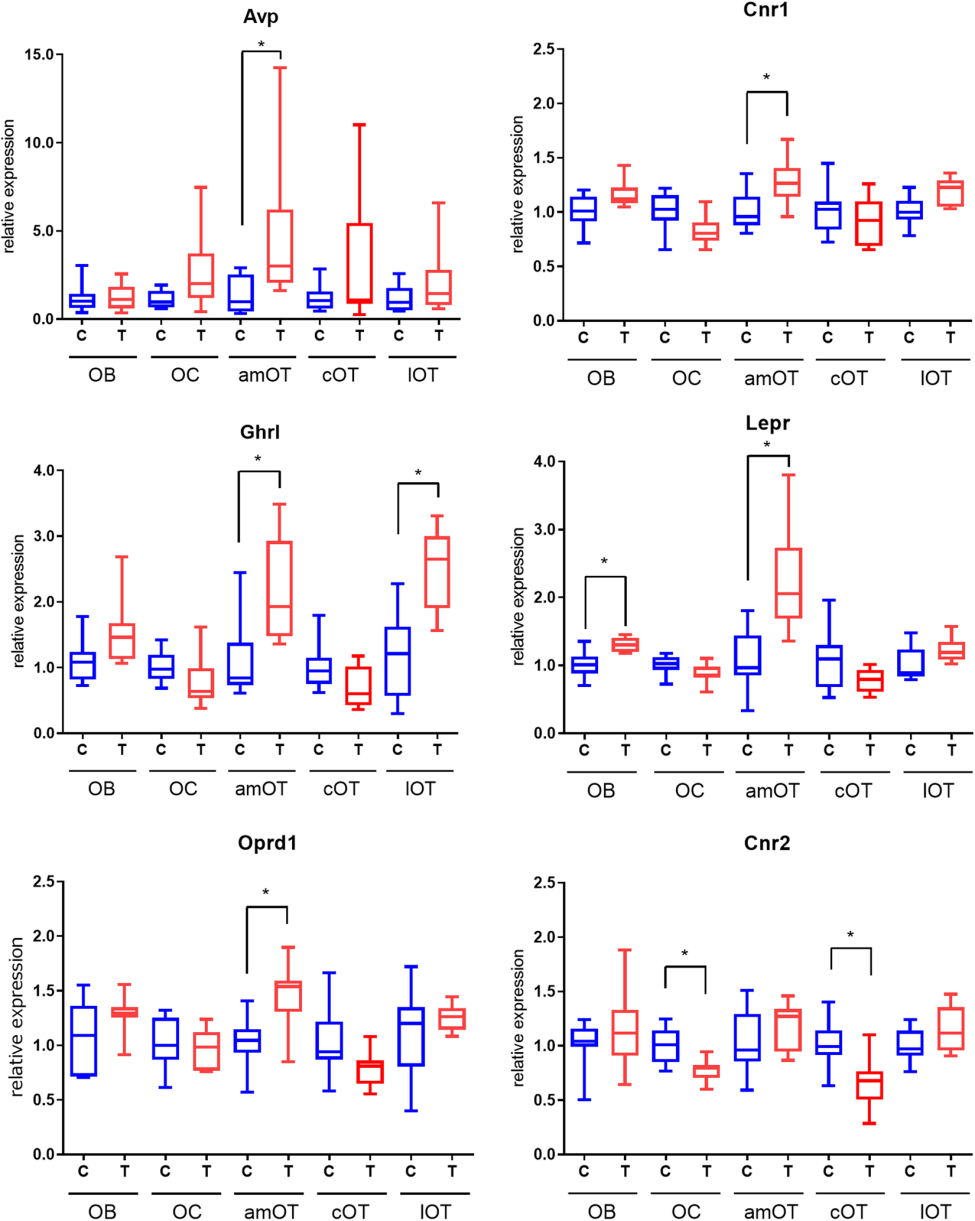
Comparison of representative neuromodulatory molecule expression in various olfactory areas between control and trained mice. The relative expression levels in control and trained mice were calculated based on the averaged ΔCt of control mice. Data for AVP, cannabinoid receptor 1 (Cnr1), ghrelin (Ghrl), leptin receptor (Lepr), opioid receptor delta 1 (Oprd1) and cannabinoid receptor 2 (Cnr2) are shown for control (blue) and trained (red) mice. Boxes indicate the 25^th^ and 75^th^ percentiles, whiskers show the minimum and maximum values, and lines inside the boxes indicate the median. *p < 0.05 (adjusted p values). n = 8–9 for each group of data.

Molecular expression in the five olfactory areas was compared between control mice and odour-food reward association-trained mice to determine whether the expression of feeding-related neuromodulatory molecules is regulated by odour-related food experience. The data were first normalized to Gapdh expression, and then evaluated using the 2^−ΔΔCT^ method^25^. The relative expression in control and trained mice (mean ± SD) was compared. Given the examination of 23 different molecules, the p values of t-test were adjusted by adopting Storey’s method for multiple comparisons^26^. Their statistical significance based on the adjusted p values are shown in Table 4 (significance, p < 0.05); representative figures are shown in Fig. 3, figures for all 23 molecules in Supplementary Fig. S2, and all the unadjusted and adjusted p values in Supplementary Table 2. In each group of data, 7–9 samples from different mice were examined.

**Table 4:** Expression of neuromodulatory molecules in individual olfactory areas of control and trained mice. Expression of each molecule was first normalized to Gapdh, and the relative values in control and trained mice were then calculated based on the averaged ΔCt of control mice. Data show the mean ± SD. Symbols indicate Storey-adjusted p values. ^#^0.05 < p < 0.1; *p < 0.05. Bold letters indicate data sets with significant p values (p < 0.05).

		OB	OC	amOT	cOT	lOT
Avp	control	1.18 ± 0.87	1.09 ± 0.64	**1.38 ± 1.28**	1.18 ± 0.86	1.20 ± 0.95
	trained	1.27 ± 0.94	2.63 ± 1.22^#^	**4.68** ± 1.02*	2.99 ± 1.72	2.05 ± 1.12
Avpr1a	control	1.03 ± 0.37	1.04 ± 0.44	1.07 ± 0.56	1.03 ± 0.38	1.04 ± 0.45
	trained	1.00 ± 0.20	0.96 ± 0.61	1.12 ± 0.47	0.97 ± 0.73	0.90 ± 0.50
Bdnf	control	1.01 ± 0.22	1.01 ± 0.23	1.20 ± 0.84	1.14 ± 0.70	1.33 ± 0.98
	trained	1.14 ± 0.12^#^	0.86 ± 0.19^#^	0.94 ± 0.48	1.51 ± 0.72	3.01 ± 1.36
Cartpt	control	1.02 ± 0.32	1.01 ± 0.25	1.01 ± 0.24	1.05 ± 0.43	1.01 ± 0.23
	trained	1.01 ± 0.13	1.15 ± 0.45	0.84 ± 0.22^#^	0.90 ± 0.16	0.98 ± 0.40
Cnr1	control	1.01 ± 0.23	1.01 ± 0.26	**1.01** ± **0.25**	1.02 ± 0.32	1.01 ± 0.19
	trained	1.16 ± 0.16^#^	0.82 ± 0.22^#^	**1.27** ± 0.21*	0.92 ± 0.34	1.18 ± 0.16^#^
Cnr2	control	1.03 ± 0.40	**1.01** ± **0.25**	1.04 ± 0.42	**1.02** ± **0.33**	1.01 ± 0.22
	trained	1.16 ± 0.44	**0.77** ± 0.19*	1.16 ± 0.27	**0.66** ± 0.54*	1.17 ± 0.26
Ghrl	control	1.10 ± 0.42	1.02 ± 0.33	**1.10** ± **0.64**	1.03 ± 0.48	**1.18** ± **0.94**
	trained	1.53 ± 0.42^#^	0.78 ± 0.64^#^	**2.16** ± 0.51*	0.70 ± 0.62^#^	**2.51** ± 0.38*
Ghsr	control	1.05 ± 0.51	1.17 ± 0.82	1.03 ± 0.34	0.67 ± 0.29	1.03 ± 0.34
	trained	1.07 ± 0.30	1.08 ± 0.98	1.14 ± 0.34	0.50 ± 0.64	1.33 ± 0.75
Hcrt	control	1.02 ± 0.34	1.01 ± 0.24	1.02 ± 0.30	1.03 ± 0.38	1.01 ± 0.22
	trained	1.05 ± 0.39	0.88 ± 0.57	1.77 ± 0.85	1.32 ± 0.98	1.35 ± 0.59
Hcrtr1	control	1.05 ± 0.49	1.02 ± 0.28	1.05 ± 0.50	1.03 ± 0.41	1.03 ± 0.35
	trained	1.02 ± 0.30	0.95 ± 0.39	1.19 ± 0.40	1.24 ± 0.48	1.36 ± 0.55
Hcrtr2	control	1.04 ± 0.40	1.01 ± 0.26	1.01 ± 0.24	1.02 ± 0.29	1.01 ± 0.19
	trained	1.18 ± 0.56	0.87 ± 0.17^#^	1.19 ± 0.29	1.12 ± 0.60	0.89 ± 0.25
Lepr	control	**1.02** ± **0.28**	1.01 ± 0.21	**1.09** ± **0.71**	1.08 ± 0.60	1.02 ± 0.32
	trained	**1.30** ± 0.11*	0.89 ± 0.24	**2.24** ± 0.46*	0.79 ± 0.34	1.22 ± 0.20^#^
Mc4r	control	1.01 ± 0.25	1.05 ± 0.47	**1.03** ± **0.34**	1.03 ± 0.36	1.01 ± 0.27
	trained	1.54 ± 0.71	0.86 ± 1.00	**1.35** ± 0.20*	0.81 ± 0.30^#^	1.24 ± 0.29^#^
Mme	control	1.04 ± 0.41	1.02 ± 0.34	**1.03** ± **0.34**	1.05 ± 0.44	1.01 ± 0.24
	trained	1.28 ± 0.14^#^	0.93 ± 0.76	**1.35** ± 0.21*	0.71 ± 0.42^#^	1.03 ± 0.36
Npy2r	control	1.12 ± 0.72	1.04 ± 0.41	1.03 ± 0.37	1.11 ± 0.70	1.03 ± 0.36
	trained	1.30 ± 0.76	0.70 ± 0.51^#^	1.22 ± 0.17	0.90 ± 0.38	1.15 ± 0.33
Oprd1	control	1.05 ± 0.48	1.03 ± 0.36	**1.03** ± **0.36**	1.04 ± 0.42	1.08 ± 0.63
	trained	1.28 ± 0.20	0.97 ± 0.28	**1.46** ± 0.34*	0.78 ± 0.30^#^	1.26 ± 0.14
Oprk1	control	1.02 ± 0.27	1.09 ± 0.65	**1.01** ± **0.19**	1.06 ± 0.49	1.01 ± 0.23
	trained	1.21 ± 0.21^#^	0.73 ± 0.73	**1.26** ± 0.21*	0.80 ± 0.30	1.06 ± 0.32
Oxt	control	1.04 ± 0.76	1.17 ± 0.84	1.38 ± 1.33	1.71 ± 1.62	1.96 ± 1.64
	trained	1.20 ± 0.77	1.77 ± 1.05	3.73 ± 1.23^#^	2.70 ± 1.45	1.98 ± 0.84
Oxtr	control	1.04 ± 0.46	**1.02** ± **0.34**	1.01 ± 0.22	1.04 ± 0.41	1.10 ± 0.71
	trained	1.27 ± 0.28	**0.67** ± 0.22*	1.13 ± 0.26	1.03 ± 0.55	0.99 ± 0.74
Pdyn	control	1.01 ± 0.26	1.02 ± 0.33	1.03 ± 0.37	1.05 ± 0.47	1.02 ± 0.34
	trained	1.18 ± 0.16^#^	0.99 ± 0.66	1.12 ± 0.20	0.72 ± 0.32^#^	0.99 ± 0.10
Penk	control	1.00 ± 0.10	1.01 ± 0.18	1.01 ± 0.17	1.05 ± 0.46	1.01 ± 0.19
	trained	1.08 ± 0.07^#^	1.12 ± 0.25	1.04 ± 0.11	0.87 ± 0.46	0.99 ± 0.21
Pomc	control	1.95 ± 2.06	2.61 ± 2.25	3.12 ± 2.54	1.47 ± 1.77	2.39 ± 2.31
	trained	0.96 ± 1.74	1.22 ± 2.37	1.50 ± 2.92	0.84 ± 2.72	0.53 ± 2.14^#^
Tac1	control	1.01 ± 0.27	1.05 ± 0.47	1.01 ± 0.23	1.04 ± 0.40	1.01 ± 0.16
	trained	0.98 ± 0.27	1.22 ± 0.99	1.03 ± 0.11	0.74 ± 0.30^#^	1.07 ± 0.18

Many molecules showed differential expression between control and trained mice, and the difference was most conspicuous in the anteromedial OT. Expression of arginine-vasopressin (AVP), cannabinoid receptor 1 (Cnr1), ghrelin (Ghrl), leptin receptor (Lepr), melanocortin 4 receptor (Mc4r), neprilysin (Mme), opioid receptor delta 1 (Oprd1) and opioid receptor kappa 1 (Oprk1) were elevated in the anteromedial OT of trained mice (Table 4, see Fig. 3 for Avp, Cnr1, Ghrl, Lepr and Oprd1 expression). Among these compounds, ghrelin (Ghrl) was also enhanced in the lateral OT (see Fig. 3 for Ghrl expression), while the other seven molecules specifically increased in the anteromedial OT among the three OT areas.

Differential expression was observed in other olfactory areas. Expression of leptin receptor (Lepr) increased in the OB of trained mice (see Fig. 3 for Lepr expression). Decreased expression in the trained mice was also observed. In the OC, cannabinoid receptor 2 (Cnr2) and oxytocin receptor (Oxtr) decreased in the trained mice. In the central OT, cannabinoid receptor 2 (Cnr2) decreased in the trained mice (see Fig. 3 for Cnr2 expression).

These results indicate that expression levels of neuromodulatory molecules in the olfactory areas were significantly altered by odour-food reward association training. The alteration was most conspicuous in the anteromedial OT, where many molecules showed increased expression in the trained mice. In other olfactory areas, not only increased expression but also decreased expression was observed in the trained mice.

While not attaining the authentic statistical significance level, some molecules showed p values below 0.1 (0.05 < p < 0.1; shown in Table 4 as #) for the differential expression in trained mice. Their alteration toward increase or decrease appeared to differ among olfactory areas. In the OB, expression of BDNF (Bdnf), cannabinoid receptor 1 (Cnr1), ghrelin (Ghrl), neprilysin (Mme), opioid receptor kappa 1 (Oprk1), prodynorphin (Pdyn) and preproenkephalin (Penk) altered toward increase in trained mice. Intriguingly, in the OC, expression of BDNF (Bdnf), cannabinoid receptor 1 (Cnr1), ghrelin (Ghrl), orexin receptor 2 (Hctr2) and neuropeptide Y receptor Y2 (Npy2r) altered toward decrease, while only arginine-vasopressin (AVP) toward increase in trained mice. In the central OT, expression of ghrelin (Ghrl), melanocortin 4 receptor (Mc4r), neprilysin (Mme), opioid receptor delta 1 (Oprd1), prodynorphin (Pdyn) and tachykinin 1 (Tac1) altered toward decrease in trained mice. In the lateral OT, cannabinoid receptor 1 (Cnr1), leptin receptor (Lepr) and melanocortin 4 receptor (Mc4r) altered toward increase and POMC (Pomc) toward decrease in trained mice. In the anteromedial OT, oxytocin (Oxt) altered toward increase and CART peptide (Cartpt) toward decrease in trained mice. The overall tendency of expression pattern may help speculating the possible roles of neuromodulatory molecules in these olfactory areas (see Discussion).

## Discussion

Here, we examined the expression of feeding-related neuromodulatory molecules in the olfactory system of food-restricted mice. While this study involved analysis of a limited number of molecules, many of them were differentially expressed in various olfactory areas and showed significantly altered expression after odour-food reward association training. This suggests that the function of the olfactory system, including its higher cortical areas, is influenced by feeding-related neuromodulatory signals and contributes to the adaptive regulation of odour-mediated feeding behaviour.

In this study, we observed that neuromodulatory molecules were more highly expressed in the OT and OC compared to the OB. Previous studies have demonstrated the effects of feeding-related molecules on OB function. Intracerebroventricular (icv) administration of the orexigenic neuropeptide orexin into rats promoted sniffing behaviour and c-fos expression in response to food odour in the OB, while icv administration of the anorexigenic compound leptin decreased these responses^27^. Application of orexin to the sliced OB potentiated odour-induced firing activity of the projecting neurons of the OB (mitral cells)^28^, while anorexigenic insulin modulated the activity of mitral cells and interneurons in the OB^29,30^. Endocannabinoid signals to the OB potentiate feeding behaviour under fasting conditions^31^. In addition to these studies of the OB, the present results suggest that functions of the OT and OC are also regulated by feeding-related neuromodulatory molecules. In fact, icv administration of insulin decreased odour responses in the pyramidal neurons of the piriform cortex^32^. Further, insulin injection into the piriform cortex prevented odour discrimination, while insulin application to a cortical slice suppressed the firing activity of piriform cortex neurons^33^. While knowledge of feeding-related signals in olfactory cortical areas remains limited, these reports, and the molecular expression results of the present study, support the hypothesis that feeding-related neuromodulatory signals strongly influence the function of olfactory cortical areas and crucially regulate odour-mediated feeding behaviours.

Numerous molecules examined in this study showed higher expression in the OT than in the OC. As expected, endogenous opioids, which are produced by dopamine receptor-expressing striatal neurons to regulate motivated and hedonic behaviours^34^, were highly expressed in the OT. In addition, opioid receptors and other feeding-related molecules showed elevated expression in the OT. Given that the OT is highly involved in motivated behaviours^16,19–21^, neuromodulatory signals likely regulate feeding behaviour by acting on the OT.

Within the OT, many feeding-related molecules were highly expressed in the anteromedial OT, representing odour-guided attractive behaviour^22^. A variety of orexigenic molecules, including orexin (Hcrt), orexin receptor 1 and 2 (Hcrtr1, 2), ghrelin receptor (Ghsr), opioid receptor kappa 1 (Oprk1) and prodynorphin (Pdyn) were more strongly expressed in the anteromedial OT compared to the lateral OT. In addition, several anorexigenic molecules, such as CART peptide (Cartpt) and melanocortin 4 receptor (Mc4r), were also highly expressed in the anteromedial OT. AVP receptor 1a, highly expressed in the anteromedial OT, is also a candidate anorexigenic molecule^35^. These observations raise the possibility that feeding-related neuromodulatory signals regulate feeding behaviours both positively and negatively, by acting on the anteromedial OT. Given the differing roles of dopamine receptor type 1 (D1)- and dopamine receptor type 2 (D2)-expressing neurons in the anteromedial OT^36^, it is essential to understand which types of OT neurons express neuromodulatory molecules. While our preliminary examination through *in situ* hybridization did not distinguish among neuron types due to the relatively low expression levels of neuromodulators in the OT (data not shown), this point needs to be addressed in future analysis. In contrast, only neprylisin (Mme), a membrane metalloendopeptidase that digests enkephalin^37^, showed higher expression in the lateral OT than in the anteromedial OT. Given that the lateral OT is linked to aversive behaviours^22^, this area might not be the major target of feeding-related neuromodulation, and may instead be influenced by fear-related neuromodulation^38^.

In odour-food association-trained mice, the expression of feeding-related neuromodulatory molecules was significantly altered. This alteration was most prominent in the anteromedial OT, among the five areas examined. In the anteromedial OT of trained mice, the expression levels of orexigenic molecules, including cannabinoid receptor 1 (Cnr1), ghrelin (Ghrl), opioid receptor delta 1 (Oprd1) and opioid receptor kappa 1 (Oprk1) increased, as did the levels of anorexigenic molecules including AVP, leptin receptor (Lepr), melanocortin 4 receptor (Mc4r) and neprilysin (Mme). These results support experience-dependent control of feeding motivation, both positively and negatively, via neuromodulation in the anteromedial OT.

While the production of neuromodulatory ligands in brain regions other than the hypothalamus is controversial^39^, training-dependent changes in the expression levels of ligands such as ghrelin (Ghrl) and AVP in the anteromedial OT might reflect their physiological roles in feeding. Given that mRNA for AVP is transported to the axon terminal^40^, the present experiments suggest the possible presence of mRNA in axons that originated from ligand-producing cells in other brain regions, such as the hypothalamus. Heterogeneous neuromodulator-producing neurons send axons into distinct brain areas^41^. The present results might represent area-specific and training-dependent ligand delivery along specific axonal trajectories. Alternatively, ligand-producing cells may be present in the olfactory area. AVP-expressing neurons are distributed in the rat OB and olfactory cortex^42,43^. Understanding the adaptive delivery of neuromodulatory ligands could reveal a crucial role of the olfactory system in controlling feeding behaviour.

In the comparison between control and trained mice, we also highlighted molecules whose altered expression showed small but not below the authentic threshold of p values, because these data help speculating the possible roles of adaptive molecular expression in the olfactory areas. Most of these cases (0.05 < adjusted p < 0.1) showed unadjusted p values below 0.05 (Supplementary Table S2). In trained mice, molecular expression in the OB tended to alter toward increase, suggesting contributions to adaptive OB function based on odour-food association. This notion is in line with many studies showing the important roles of neuromodulators in the OB for adaptive feeding behaviour^13,14,31^. Intriguingly, molecular expression in the trained mice tended to alter toward decrease in the central OT and OC, hinting at currently unknown roles of the central OT in motivated behaviours, and differential roles of the OT and OC in the control of feeding behaviours.

One limitation of the present study is that a limited number of molecules among a vast repertoire of feeding-related molecules were examined. The tendencies observed may not be applicable to molecules that were not examined directly in this study. Secondly, we did not address the possible influence of nutritional state on the molecular expression. We here focused our analysis on food-restricted mice to effectively address the contribution of odour-association training. Expression of feeding-related molecules can be influenced by the hunger-satiety state, as shown for the receptors of leptin and neuropeptide Y in the olfactory epithelium^44,45^. While we controlled the body weight of control and trained mice at the same level and we did not observe difference in the glucose or insulin contents between the two groups of mice, influence of sugar supply to trained mice on the molecular expression cannot be completely denied. Possible alteration of molecular expression in olfactory cortical areas depending on the nutritional state must be addressed in further experiments. Thirdly, molecular expression was evaluated only through PCR. Due to the relatively low expression levels of neuromodulatory molecules in the olfactory system, the spatial distribution and types of cells expressing these molecules remain unclear. While we carefully dissected olfactory cortical areas, inclusion of adjacent structures such as anterior ventral pallidum, which may share functions with the OT, in the OT samples cannot be avoided. Detailed histological analysis *in situ* would supplement the current observation. Nonetheless, the present analysis shows differential expression of numerous feeding-related neuromodulatory molecules among different olfactory areas, and regulation of their expression by odour-food association training. This fundamental knowledge will facilitate investigation of the activities of individual neuromodulatory molecules in the olfactory system, and their contributions to adaptive regulation of odour-mediated feeding behaviour.

## Methods

### Animals

All experiments were conducted in accordance with the guidelines of the Physiological Society of Japan and were approved by the Kochi Medical School Animal Care and Use Committee. Male C57BL/6 mice (Japan SLC Inc., Sizuoka, Japan) were housed individually in plastic cages (24 × 17 × 12 cm) with wood shavings at 26 °C under a 12-h light/dark cycle (light on at 21:00 and off at 9:00). Odour-food association training was initiated when mice were 8 weeks of age.

### Odour-food association training and behavioural assay

For odour and sugar association training, mice were food-restricted to achieve 80–90% of their *ad libitum* feeding body weight. Food pellets were removed 2 days prior to the start of odour-sugar association training. A limited amount of food pellets (2.7–3.3 g per day) was then delivered, to maintain 80–90% body weight during the training period. Water was available *ad libitum* throughout the experiment. Association training and behavioural tests were conducted during dark phase (between 9:00 and 12:00; dark phase was 9:00–21:00). Association training, of odour with the sugar reward, was conducted in a plastic conditioning cage (24 × 17 × 12 cm) with 2-cm-deep paper bedding (Japan SLC Inc.). During the first trial on day 1, mice learned to eat sugar; they were presented with sugar (granulated sucrose; 20–40 mg) together with powdered diet (20–40 mg) on a petri dish, with holes exposing a filter paper (2 cm × 2 cm) soaked with 10 µl of eugenol (Tokyo Chemical Industry, Tokyo, Japan) in the lower compartment. In the three successive trials of day 1, the mice underwent association training with the sugar reward (50 mg; without powdered diet) and eugenol in the same dish. The dish was hidden under the bedding (2 cm depth) so that the mouse could locate it by smell, and the position of the hidden dish within the conditioning cage was randomly chosen. Because mice could find and eat the sugar within 2 min, the duration of one trial was fixed at 2 min. Four training trials per day were conducted for a further 7 consecutive days (days 2–8). For control mice, the same food restriction was applied, and a holed petri dish containing filter paper soaked with 10 µl of eugenol without sugar was presented for one 2-min trial. Four trials per day were conducted from day 1 to day 8. For the first and fourth trials on day 8, the latency period to the start of digging over the dish with eugenol (with or without sugar) was measured for randomly sampled trained and control mice. Controlled amount of food pellet was delivered after the each day’s training.

On day 9, mice were habituated to a larger test cage (30 × 20 × 13 cm) with paper bedding (2 cm depth) for 30 min. Then, a dish containing eugenol, but without sugar, was buried under the bedding of the test cage and presented to both trained and control mice. The behaviour of sampled mice for 30 min after odour presentation was video-recorded, and the total duration of digging behaviour over the scented dish over 30 min was evaluated.

### Dissection of olfactory areas in the brain

Mice were deeply anesthetized through intraperitoneal injection of sodium pentobarbital. They were transcardially perfused with phosphate-buffered saline (PBS) and then decapitated. The brain was removed from the skull, and the OB, amOT, lOT, cOT and OC, aside from the OT (major structure was likely the piriform cortex), were dissected and collected separately. The boundary between the amOT and cOT was a line extending mediocaudally from the rostral pole of the OT to separate semicircular anteromedial OT region, and that between the cOT and lOT was a line connecting the rostral and lateral poles of the OT (see Fig. 1C). The OC was dissected from the lateral and caudolateral area to the OT, including the piriform cortex.

For quantitative real-time PCR analysis, each brain tissue specimen was soaked in 1.5 ml of RNAlater solution (Thermo Fisher Scientific, Waltham, MA, USA) for at least 8 h at 4 °C, and then held at −80 °C in the same solution until analysis. For western blot analysis, the OB, OT (recovered as one block without separation), OC, and dorsal area of the neocortex (major structure was likely the somatosensory cortex) were recovered. These specimens were soaked in protein solution, as described in the following section.

### Histological analysis

After dissection of the olfactory areas, the brain was immersed in 4% paraformaldehyde in 0.1 M phosphate buffer (PB) overnight, and then transferred to 30% sucrose in 0.1 M PB. The brain was then embedded in optimal cutting temperature (OCT) compound (Sakura Finetechnical, Tokyo, Japan), frozen at −80 °C, and sliced into coronal sections with a thickness of 50 μm using a cryotome. The sections were rinsed with PBS, mounted on slide glasses, and stained with DAPI (1 µg/ml) for 5 min. After washing in PBS, sections were mounted in Prolong Gold (Thermo Fisher Scientific). The intact brain without olfactory area dissection was processed using the same procedure. Images were acquired using fluorescence microscopy.

### Western blotting

Dissected brain tissues were soaked in lysis buffer containing 1× RIPA buffer (Cat# 89900; Thermo Fisher Scientific), 0.5 M EDTA and 1× protease and phosphatase inhibitor cocktail (Cat# 78440; Thermo Fisher Scientific) at 4 °C. The tissues were sonixged at 14,000 g for 20 min at 4 °C; their supernatants were then collected. Brain extracts containing 20 µg protein were fractionated through SDS–PAGE using a polyacrylamide gel (Cat# 4561096; Bio-Rad, Hercules, CA, USA) according to the manufacturer’s protocol. The proteins were transferred to polyvinylidene fluoride (PVDF) membranes blocked with 4% skim milk in TTBS (in mM: 150 NaCl, 20 Tris, pH 7.5, 0.1% Tween-20) overnight at 4 °C, and incubated with a primary antibody, anti-DARPP32 (1:400; Cat# ab40801; Abcam, Cambridge, UK) or anti-RGS14 (1:500; Cat# GTX15147; GeneTex, Irvine, CA, USA), for 1 h at room temperature. After three rinses with TTBS for 5 min, the membranes were incubated with the secondary antibody, horseradish peroxidase (HRP)-conjugated anti-rabbit IgG (1:5000, Cat# 401315; Millipore, Billerica, MA, USA), for 30 min at room temperature. After three rinses with TTBS for 5 min, the blots were developed with Luminata^TM^ Forte Western HRP Substrate (Millipore) and the Lumino Image Analyzer System LAS4000 (Fujifilm, Tokyo, Japan).

### Measurement of blood glucose and plasma insulin concentrations

Different sets of mice were similarly food-restricted and received odour-food association procedure. After the behavioural test on day 9, they were deeply anesthetized through intraperitoneal injection of sodium pentobarbital. Then the thoracic cavity was opened, the body fluid in the cavity was removed by using cotton bud, and the right atrium of the heart was cut by scissors. The blood leaked into the thoracic cavity was recovered and measured for glucose concentration by using a blood glucose meter (FreeStyle, Abbott, Canada). For the measurement of insulin concentrations, the blood was collected in a tube containing EDTA (final concentration, 1 mg/ml). The samples were ice-cooled and centrifuged at 1,800 g for 10 min at 4 °C, and the plasma was collected. The plasma insulin concentrations were determined using a Mouse/Rat Insulin ELISA kit (Ultra sensitive kit, Morinaga Institute of Biological Science, Japan) according to the manufacturer’s instruction.

### RNA extraction and quantification

The corresponding right and left olfactory areas of each mouse brain were collected as one tissue sample. Total RNA from each tissue sample was extracted using the RNeasy Lipid Tissue Mini Kit (Qiagen Ltd., Tokyo, Japan). The tissue samples were placed into 200 μl of QIAzol Lysis Reagent, homogenized with a disposable pestle (Sansyo Co., Ltd., Tokyo, Japan), and combined with 800 μl of QIAzol Lysis Reagent. Then, the samples were processed using the RNeasy Lipid Tissue kit, following the manufacturer’s instructions. On-column DNase digestion (RNase-Free DNase Set; Qiagen Ltd.) was conducted to remove genomic DNA.

The RNA from OC and OB samples was eluted with 30 μl of nuclease-free water, and that from OT samples was eluted with 20 μl of nuclease-free water. The quantity and quality of the extracted RNA was measured using a Nanodrop spectrophotometer (Thermo Fisher Scientific) and Agilent 2100 Bioanalyzer (Agilent Technologies. Ltd., Santa Clara, CA, USA). The OC and OB samples were evaluated with the Agilent RNA 6000 Nano Kit, and OT samples with the Agilent RNA 6000 Pico Kit. Only RNA samples with an RNA integrity number (RIN) of at least 7.5 were used in this study. When needed, RNA was concentrated using a SpeedVac (Thermo Fisher Scientific) to achieve a final concentration of 12.5 ng/μl.

### cDNA synthesis

cDNA was synthesized using SuperScript™ IV VILO™ Master Mix (Thermo Fisher Scientific) according to a modified version of the manufacturer’s instructions. First, 2 μl of SuperScript™ IV VILO™ Master Mix was added to 8 μl of extracted RNA (100 ng total RNA) in reaction tubes. Reverse transcription was conducted by heating the reaction mixture to 25 °C for 10 min, 50 °C for 10 min, and 85 °C for 5 min, before cooling to 4 °C with the Applied Biosystems GeneAmp PCR system 9700 (Thermo Fisher Scientific). As the majority of samples were small, the minimum amount of cDNA that could be detected by PCR probes was determined in advance, as was the dilution concentration. Synthesized cDNA was diluted 10-fold with 10 mM Tris-HCl (pH 8.0) and stored at −70 °C.

### Quantitative-PCR (q-PCR)

The q-PCR assay was performed using a QuantStudio 5 Real-time PCR system (Thermo Fisher Scientific) according to the manufacturer’s instructions. The reaction was carried out with TaqMan™ Gene Expression Master Mix (Thermo Fisher Scientific) in a 20-μl volume containing 2 μl of cDNA. The protocol took approximately 60 min to complete, including a 2-min incubation step at 50 °C and an enzyme activation step for 10 min at 95 °C, followed by 40 15-s cycles of denaturation at 95 °C and an annealing and extension step for 1 min at 60 °C. Reactions were performed in duplicate and the threshold cycle (Ct) values were averaged among replicates. Negative controls were included to detect possible contamination. The q-PCR probes used are listed in Table 1.

### Data analysis

An amplification curve was generated after analysing the raw data and adjusting the Ct values. Glyceraldehyde 3-phosphate dehydrogenase (Gapdh) was used as the internal control gene for normalizing the relative expression levels of genes of interest. Relative expression was evaluated using 2^−ΔCT^ for Tables 2, 3, Fig. 2 and Supplementary Fig. S1, and the relative expression in control and trained mice was compared using the 2^−ΔΔCT^ method^25^ for Table 4, Fig. 3, Supplementary Fig. 2 and Table 2. In the 2^−ΔΔCT^ analysis, the average of ΔCT values in control mice was determined and the ΔΔCT value in each control and trained mouse was caleraged ΔCT of control mice. Then the 2^−ΔΔCT^ values were calculated and subjected to statistical analyses.

### Statistics

Statistical analyses were performed using R software (version 3.4.3; R Core Team, 2017). One-way ANOVA, followed by Tukey-Kramer’s multiple comparison test (expression in control mice relative to Gapdh gene expression) was used for comparisons among the five olfactory areas. *P* values for the comparisons between control and trained mice were calculated using a two-tailed Student’s t-test. The p values were further adjusted by using Storey’s method for multiple comparisons^26^. Statistical significance was set at p < 0.05. Graphs were constructed using GraphPad Prism software (version 8.0; GraphPad Software Inc., La Jolla, CA, USA). In the box plot, the box indicates the 25^th^ and 75^th^ percentiles, defined as the interquartile range (IQR), while whiskers indicate the minimum and maximum values, and the line inside the box represents the median.

## Supplementary information

Supplementary Information.

## Data Availability

In publication we make materials, data and associated protocols promptly available to readers without undue qualifications in material transfer agreements.
