# Supplementary material for: Expression of feeding-related neuromodulatory signalling molecules in the mouse central olfactory system
Source: Sci Rep. 2020 Jan 21;10:890. doi: 10.1038/s41598-020-57605-7 (PMC6972952; doi:10.1038/s41598-020-57605-7)

**Supplementary Information**

# Expression of feeding-related neuromodulatory signalling molecules in the mouse central olfactory system

*Yasuko Nogi[1], *Md Monjurul Ahasan[2], Yoshihiro Murata[2], Mutsuo Taniguchi[2], Md Fazley Rabbi Sha[2], Chiori Ijichi[1], Masahiro Yamaguchi[2]

[1]Institute of Food Sciences and Technologies, Ajinomoto Co., Inc., Kanagawa, Japan

[2]Department of Physiology, Kochi Medical School, Kochi University, Kochi, Japan

Supplementary Table S1. *F* and *p* values for molecular expression in five olfactory areas.

n = 7-9 for each area (one-way ANOVA)

| genes | *F* and degrees of freedom | *p* |
|---|---|---|
| Avp | $F_{(4,40)} = 10.28$ | $p < 0.0001$ |
| Avpr1a | $F_{(4,40)} = 31.29$ | $p < 0.0001$ |
| Bdnf | $F_{(4,40)} = 242.89$ | $p < 0.0001$ |
| Cartpt | $F_{(4,40)} = 116.44$ | $p < 0.0001$ |
| Cnr1 | $F_{(4,40)} = 47.41$ | $p < 0.0001$ |
| Cnr2 | $F_{(4,40)} = 29.31$ | $p < 0.0001$ |
| Ghrl | $F_{(4,38)} = 16.95$ | $p < 0.0001$ |
| Ghsr | $F_{(4,38)} = 11.60$ | $p < 0.0001$ |
| Hcrt | $F_{(4,40)} = 9.76$ | $p < 0.0001$ |
| Hcrtr1 | $F_{(4,40)} = 35.28$ | $p < 0.0001$ |
| Hcrtr2 | $F_{(4,40)} = 64.09$ | $p < 0.0001$ |
| Lepr | $F_{(4,40)} = 5.51$ | $p = 0.0013$ |
| Mc4r | $F_{(4,40)} = 74.96$ | $p < 0.0001$ |
| Mme | $F_{(4,40)} = 67.39$ | $p < 0.0001$ |
| Npy2r | $F_{(4,40)} = 21.53$ | $p < 0.0001$ |
| Oprd1 | $F_{(4,40)} = 20.59$ | $p < 0.0001$ |
| Oprk1 | $F_{(4,40)} = 59.65$ | $p < 0.0001$ |
| Oxt | $F_{(4,38)} = 4.05$ | $p = 0.0079$ |
| Oxtr | $F_{(4,40)} = 42.47$ | $p < 0.0001$ |
| Pdyn | $F_{(4,40)} = 58.41$ | $p < 0.0001$ |
| Penk | $F_{(4,40)} = 58.86$ | $p < 0.0001$ |
| Pomc | $F_{(4,35)} = 0.80$ | $p = 0.5351$ |
| Tac1 | $F_{(4,40)} = 81.00$ | $p < 0.0001$ |

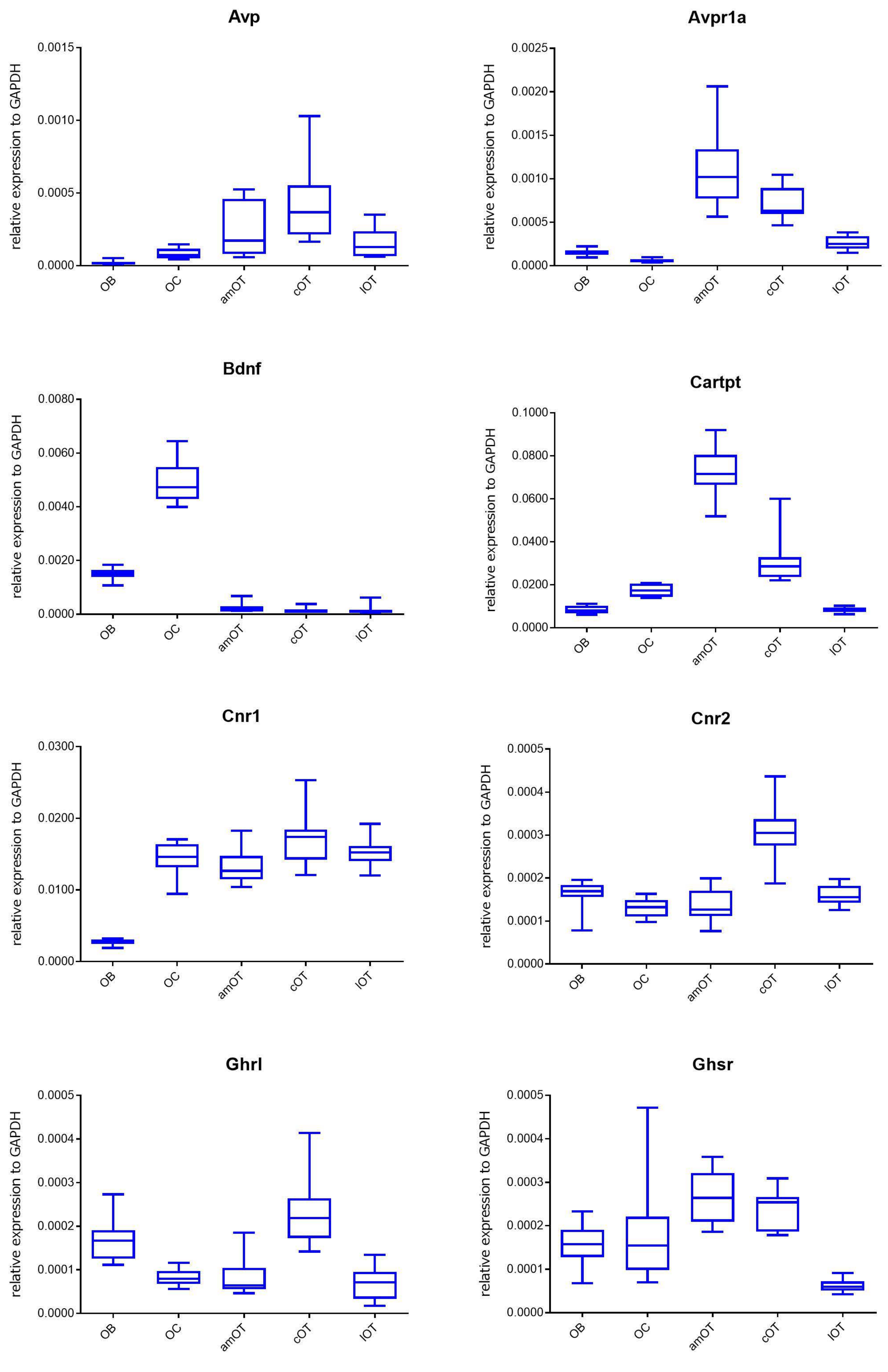

Avp
Avpr1a
Bdnf
Cartpt
Cnr1
Cnr2
Ghrl
Ghsr
relative expression to GAPDH
0.0000
0.0005
0.0010
0.0015
0.0020
0.0025
0.0040
0.0060
0.0080
0.0200
0.0300
0.0400
0.0600
0.0800
0.1000
0.0100
0.0001
0.0002
0.0003
0.0004
OB
OC
amOT
cOT
lOT

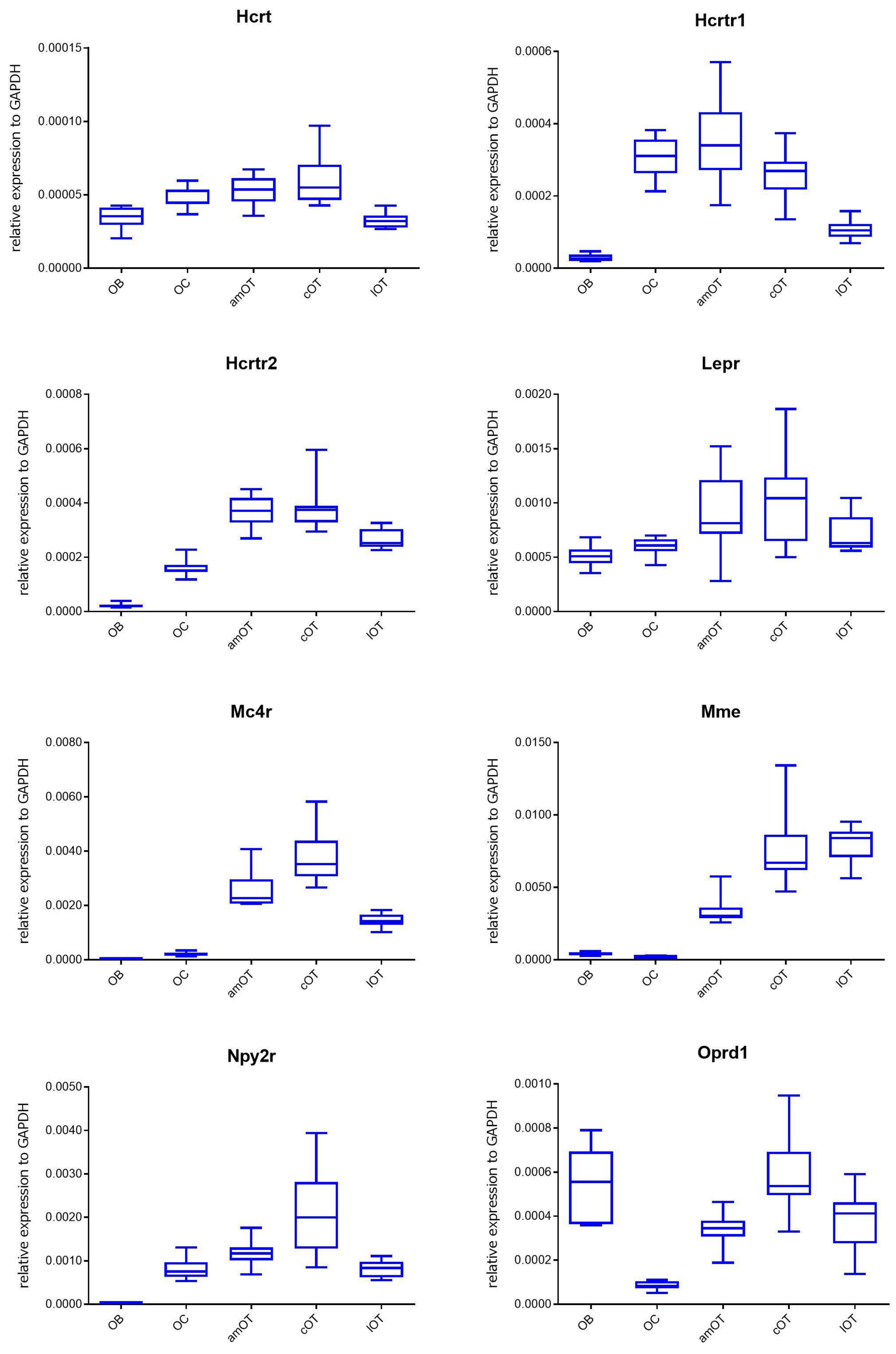

Hcrt
relative expression to GAPDH
0.00015
0.00010
0.00005
0.00000
OB
OC
amOT
cOT
lOT
Hcrtr1
relative expression to GAPDH
0.0006
0.0004
0.0002
0.0000
OB
OC
amOT
cOT
lOT
Hcrtr2
relative expression to GAPDH
0.0008
0.0006
0.0004
0.0002
0.0000
OB
OC
amOT
cOT
lOT
Lepr
relative expression to GAPDH
0.0020
0.0015
0.0010
0.0005
0.0000
OB
OC
amOT
cOT
lOT
Mc4r
relative expression to GAPDH
0.0080
0.0060
0.0040
0.0020
0.0000
OB
OC
amOT
cOT
lOT
Mme
relative expression to GAPDH
0.0150
0.0100
0.0050
0.0000
OB
OC
amOT
cOT
lOT
Npy2r
relative expression to GAPDH
0.0050
0.0040
0.0030
0.0020
0.0010
0.0000
OB
OC
amOT
cOT
lOT
Oprd1
relative expression to GAPDH
0.0010
0.0008
0.0006
0.0004
0.0002
0.0000
OB
OC
amOT
cOT
lOT

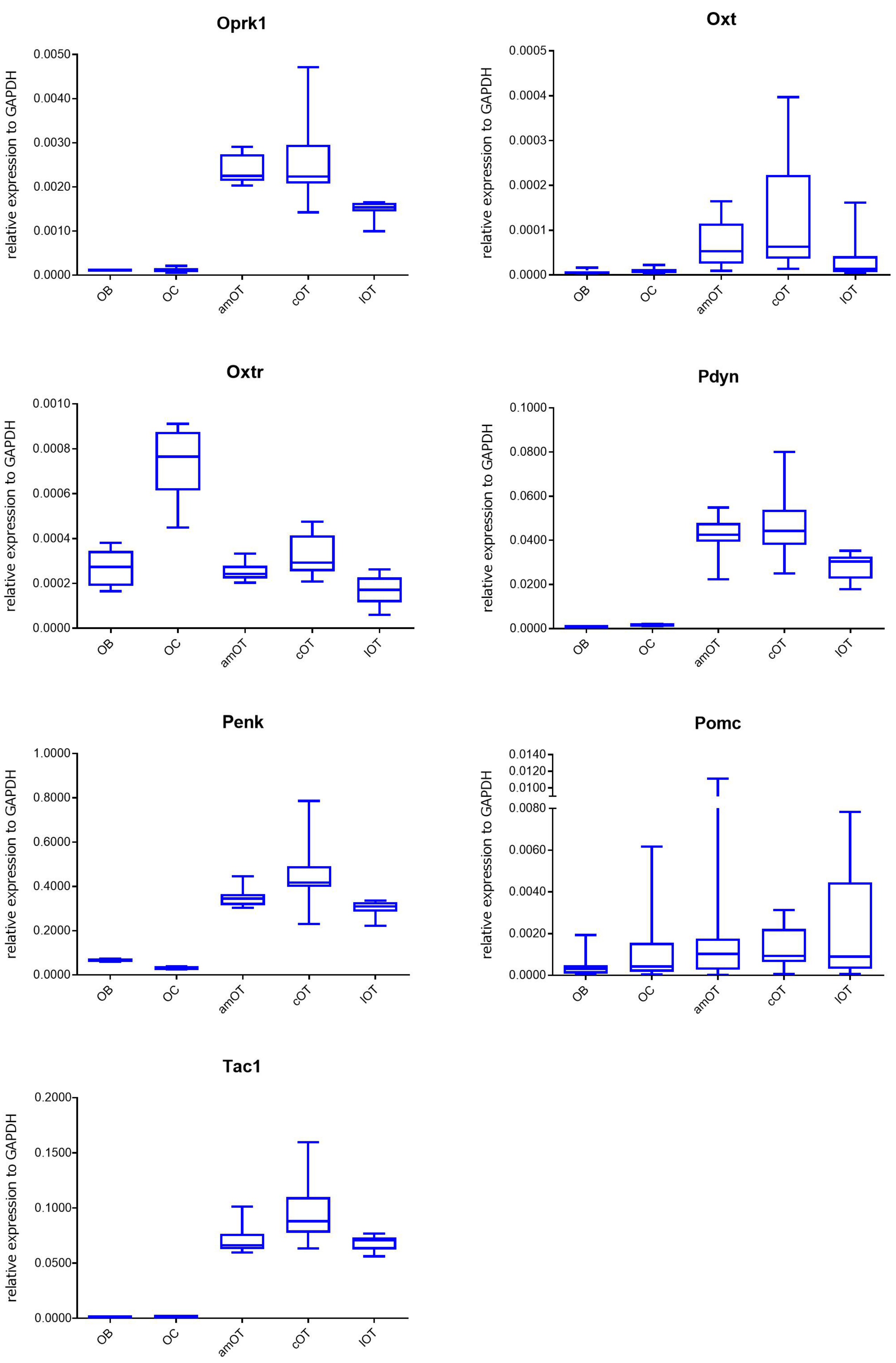


**Supplementary Figure S1.**

**Boxplot analysis of the expression of all neuromodulatory molecules in various olfactory areas.**

Boxes indicate the 25th and 75th percentiles, whiskers show the minimum and maximum values, and lines inside the boxes indicate the median. n = 7 - 9 for all molecules.

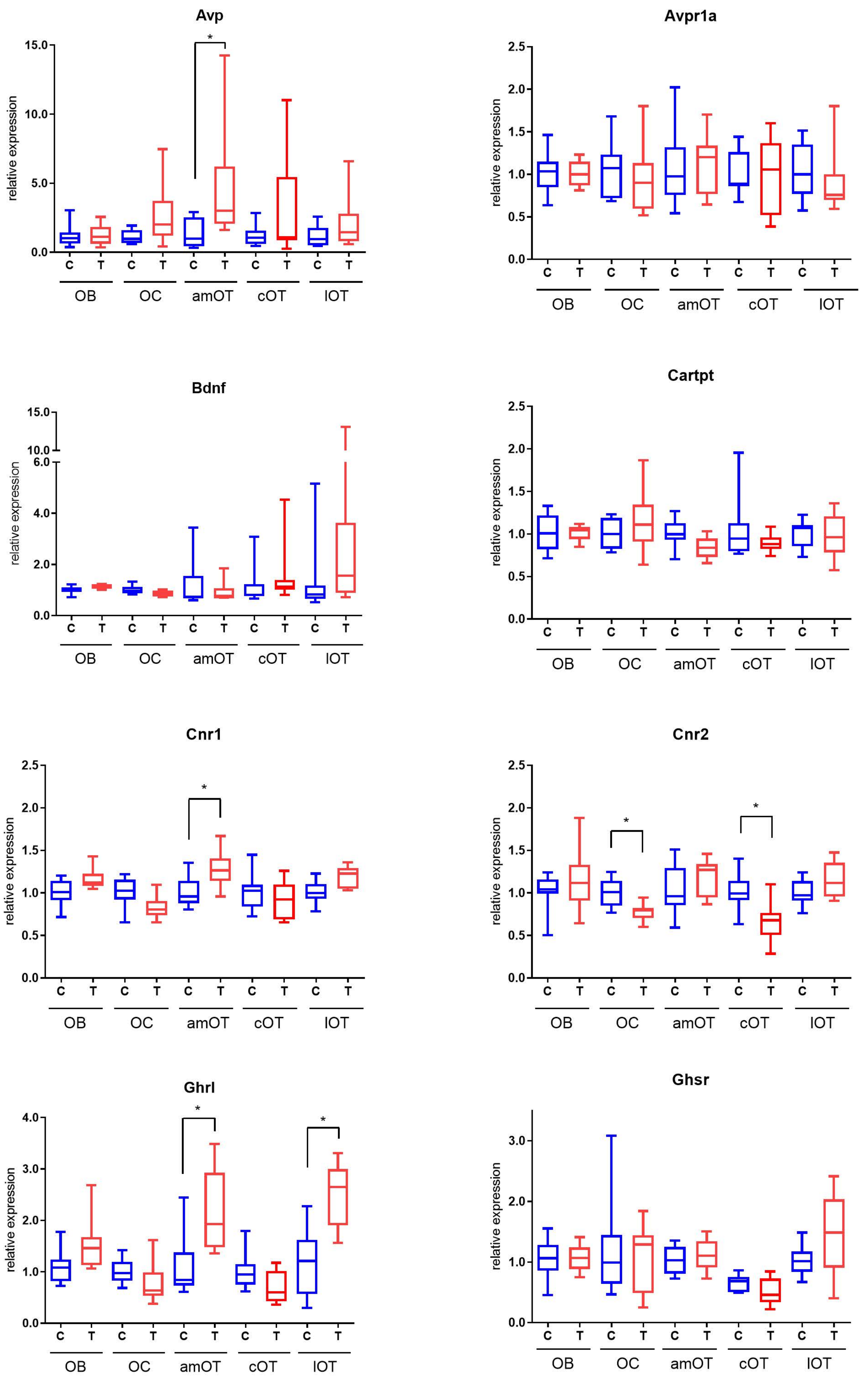
Avp
Avpr1a
Bdnf
Cartpt
Cnr1
Cnr2
Ghrl
Ghsr
relative expression
*
C
T
OB
OC
amOT
cOT
lOT
0.0
5.0
10.0
15.0
0.5
1.0
1.5
2.0
2.5
4.0
6.0
3.0

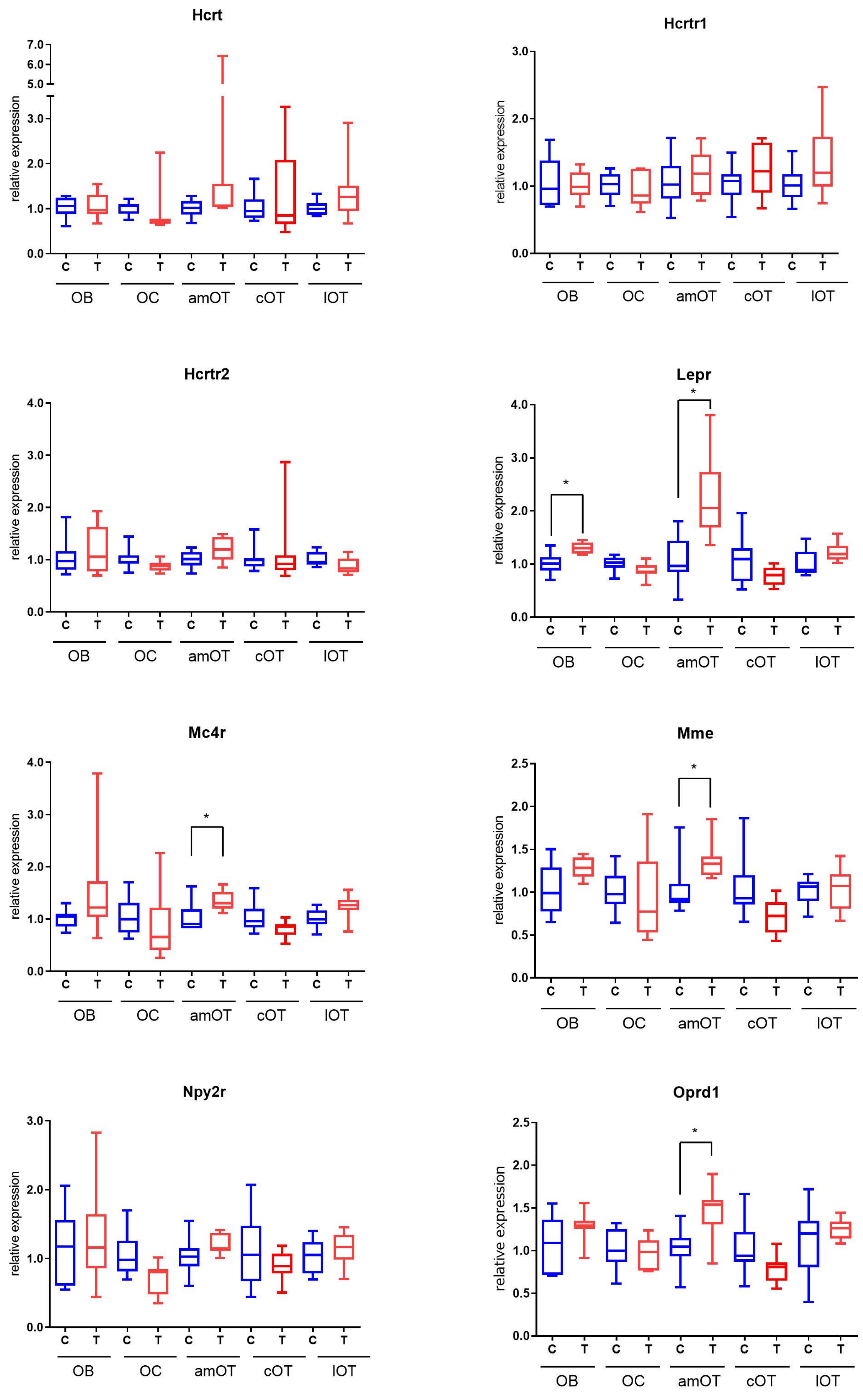

Hcrt
relative expression
7.0
6.0
5.0
3.0
2.0
1.0
0.0
C T C T C T C T C T
OB OC amOT cOT lOT
Hcrtr1
relative expression
3.0
2.0
1.0
0.0
C T C T C T C T C T
OB OC amOT cOT lOT
Hcrtr2
relative expression
4.0
3.0
2.0
1.0
0.0
C T C T C T C T C T
OB OC amOT cOT lOT
Lepr
relative expression
4.0
3.0
2.0
1.0
0.0
*
*
C T C T C T C T C T
OB OC amOT cOT lOT
Mc4r
relative expression
4.0
3.0
2.0
1.0
0.0
*
C T C T C T C T C T
OB OC amOT cOT lOT
Mme
relative expression
2.5
2.0
1.5
1.0
0.5
0.0
*
C T C T C T C T C T
OB OC amOT cOT lOT
Npy2r
relative expression
3.0
2.0
1.0
0.0
C T C T C T C T C T
OB OC amOT cOT lOT
Oprd1
relative expression
2.5
2.0
1.5
1.0
0.5
0.0
*
C T C T C T C T C T
OB OC amOT cOT lOT

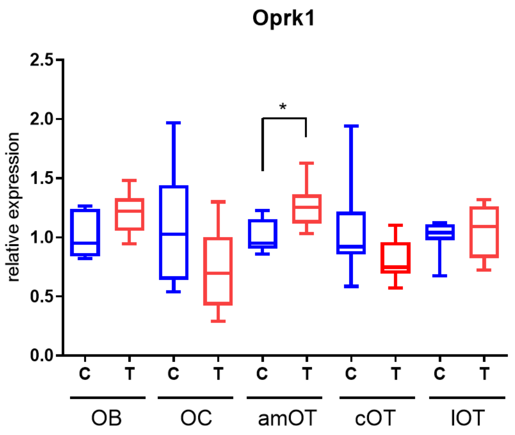


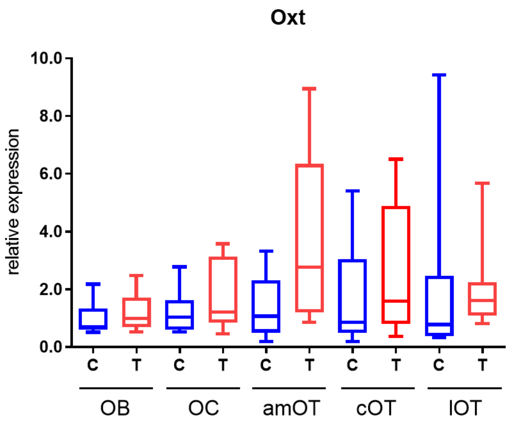


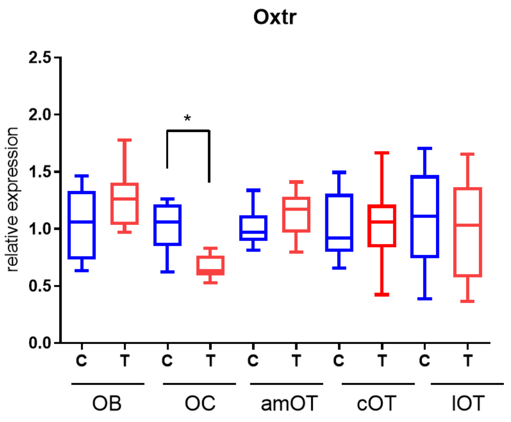


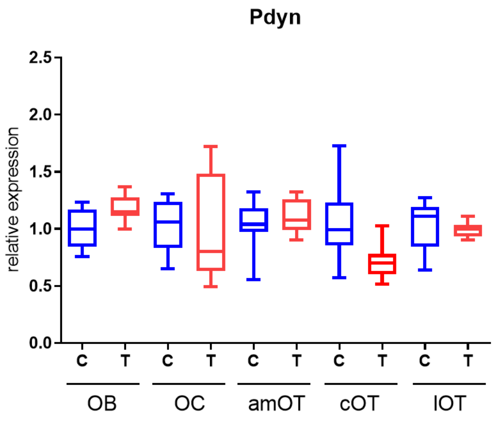


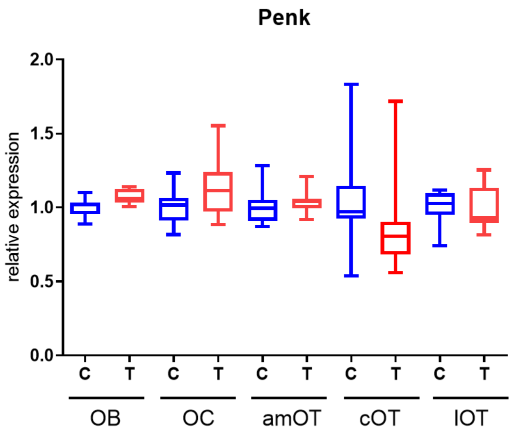


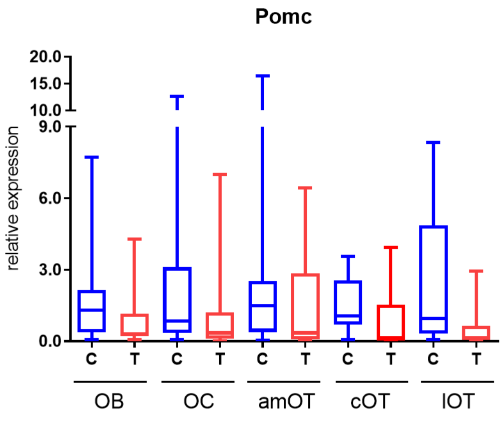


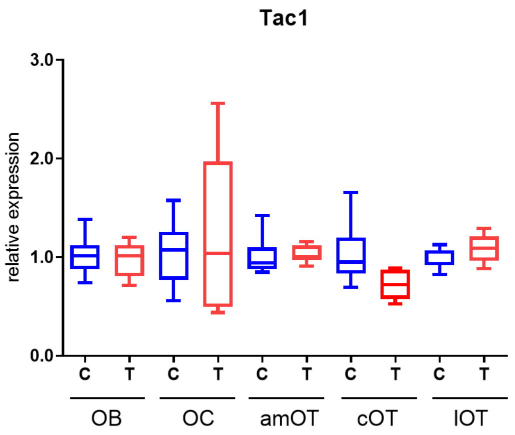


**Supplementary Figure S2.**
**Comparison of the expression of all neuromodulatory molecules in various olfactory areas between control and trained mice.**

The relative expression levels in control and trained mice were calculated based on the averaged ΔCt of control mice. Data for control (blue) and trained (red) mice are shown.
Boxes indicate the 25th and 75th percentiles, whiskers show the minimum and maximum values, and lines inside the boxes indicate the median.
*, $p < 0.05$ (Storey-adjusted p values).
n = 7 - 9 for all molecules.

Supplementary Table S2. P values for the comparisons of molecular expression between control and trained mice.

Unadjusted p values of t-test and Storey-adjusted p values are indicated.

Bold-black letters indicate $p < 0.05$. Plain-black letters indicate $0.05 < p < 0.1$ for adjusted p values. Gray letters indicate higher p values.

Upward arrows indicate increased expression and downward arrows decreased expression in trained mice.

Bold arrows with gray background represent data whose adjusted p values are below 0.05.

Plain arrows represent data whose adjusted p values are $0.05 < p < 0.1$.

| | OB | | | OC | | | amOT | | | cOT | | | lOT | | |
|---|---|---|---|---|---|---|---|---|---|---|---|---|---|---|---|
| | unadjusted | adjusted | | unadjusted | adjusted | | unadjusted | adjusted | | unadjusted | adjusted | | unadjusted | adjusted | |
| Avp | 0.824 | 0.526 | | **0.049** | 0.093 | ↑ | **0.003** | **0.028** | **↑** | 0.298 | 0.266 | | 0.232 | 0.247 | |
| Avpr1a | 0.925 | 0.559 | | 0.482 | 0.368 | | 0.711 | 0.477 | | 0.499 | 0.376 | | 0.307 | 0.271 | |
| Bdnf | **0.042** | 0.090 | ↑ | **0.036** | 0.089 | ↓ | 0.603 | 0.432 | | 0.276 | 0.261 | | 0.134 | 0.165 | |
| Cartpt | 0.985 | 0.579 | | 0.408 | 0.329 | | **0.027** | 0.084 | ↓ | 0.295 | 0.266 | | 0.612 | 0.432 | |
| Cnr1 | **0.040** | 0.090 | ↑ | **0.020** | 0.066 | ↓ | **0.007** | **0.038** | **↑** | 0.327 | 0.284 | | **0.014** | 0.057 | ↑ |
| Cnr2 | 0.463 | 0.361 | | **0.002** | **0.019** | **↓** | 0.275 | 0.261 | | **0.005** | **0.036** | **↓** | 0.084 | 0.126 | |
| Ghrl | **0.036** | 0.089 | ↑ | 0.056 | 0.099 | ↓ | **0.002** | **0.019** | **↑** | **0.046** | 0.091 | ↓ | **0.002** | **0.019** | **↑** |
| Ghsr | 0.710 | 0.477 | | 0.769 | 0.501 | | 0.365 | 0.298 | | 0.073 | 0.119 | | 0.350 | 0.293 | |
| Hcrt | 0.874 | 0.545 | | 0.135 | 0.165 | | 0.118 | 0.152 | | 0.872 | 0.545 | | 0.150 | 0.177 | |
| Hcrtr1 | 0.993 | 0.579 | | 0.447 | 0.352 | | 0.357 | 0.295 | | 0.258 | 0.261 | | 0.131 | 0.165 | |
| Hcrtr2 | 0.553 | 0.408 | | 0.056 | 0.099 | ↓ | 0.080 | 0.122 | | 0.919 | 0.559 | | 0.094 | 0.126 | |
| Lepr | **0.002** | **0.019** | **↑** | 0.092 | 0.126 | | **0.001** | **0.019** | **↑** | 0.117 | 0.152 | | **0.042** | 0.090 | ↑ |
| Mc4r | 0.089 | 0.126 | | 0.174 | 0.198 | | **0.006** | **0.036** | **↑** | 0.052 | 0.097 | ↓ | **0.043** | 0.090 | ↑ |
| Mme | **0.029** | 0.084 | ↑ | 0.289 | 0.265 | | **0.006** | **0.036** | **↑** | **0.015** | 0.057 | ↓ | 0.983 | 0.579 | |
| Npy2r | 0.577 | 0.421 | | **0.015** | 0.057 | ↓ | 0.058 | 0.100 | | 0.482 | 0.368 | | 0.330 | 0.284 | |
| Oprd1 | 0.064 | 0.107 | | 0.689 | 0.471 | | **0.006** | **0.036** | **↑** | **0.036** | 0.089 | ↓ | 0.147 | 0.177 | |
| Oprk1 | **0.037** | 0.089 | ↑ | 0.080 | 0.122 | | **0.003** | **0.028** | **↑** | 0.090 | 0.126 | | 0.726 | 0.479 | |
| Oxt | 0.619 | 0.432 | | 0.275 | 0.261 | | **0.028** | 0.084 | ↑ | 0.258 | 0.261 | | 0.251 | 0.261 | |
| Oxtr | 0.092 | 0.126 | | **0.000** | **0.019** | **↓** | 0.189 | 0.211 | | 0.878 | 0.545 | | 0.618 | 0.432 | |
| Pdyn | **0.036** | 0.089 | ↑ | 0.533 | 0.397 | | 0.286 | 0.265 | | **0.017** | 0.059 | ↓ | 0.892 | 0.549 | |
| Penk | **0.015** | 0.057 | ↑ | 0.163 | 0.188 | | 0.441 | 0.352 | | 0.219 | 0.241 | | 0.803 | 0.518 | |
| Pomc | 0.336 | 0.285 | | 0.980 | 0.579 | | 0.270 | 0.261 | | 0.080 | 0.122 | | **0.044** | 0.090 | ↓ |
| Tac1 | 0.729 | 0.479 | | 0.769 | 0.501 | | 0.647 | 0.448 | | **0.013** | 0.057 | ↓ | 0.265 | 0.261 | |

**Images of immunoblots for Figure 1E**

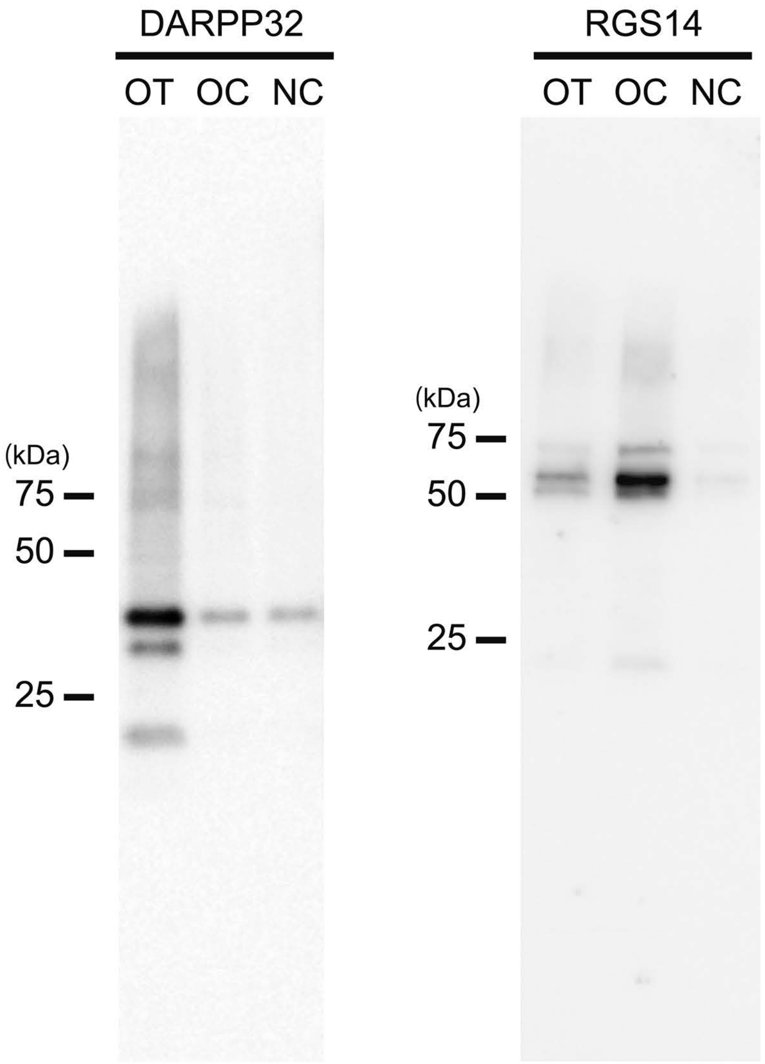

Supplement: Supplementary file 1 — Supplementary Information. [file 41598_2020_57605_MOESM1_ESM.pdf]
